# Association of *ARRDC3* and *NFIA* variants with bovine congestive heart failure in feedlot cattle

**DOI:** 10.12688/f1000research.109488.2

**Published:** 2024-03-04

**Authors:** Michael P. Heaton, Gregory P. Harhay, Adam S. Bassett, Halden J. Clark, Jaden M. Carlson, Erin E. Jobman, Helen R. Sadd, Madeline C. Pelster, Aspen M. Workman, Larry A. Kuehn, Theodore S. Kalbfleisch, Heather Piscatelli, Michael Carrie, Greta M. Krafsur, Dale M. Grotelueschen, Brian L. Vander Ley

**Affiliations:** 1 USDA, ARS, US Meat Animal Research Center, Clay Center, Nebraska, 68933, USA; 2 University of Nebraska-Lincoln, Great Plains Veterinary Educational Center, Clay Center, Nebraska, 68933, USA; 3 University of Kentucky, Lexington, Kentucky, 40506, USA; 4 MatMaCorp, Lincoln, Nebraska, 68507, USA; 5 Anschutz Medical Campus, University of Colorado Denver, Aurora, Colorado, 80045, USA

**Keywords:** Bovine, Congestive Heart Failure, ARRDC3, NFIA, GWAS

## Abstract

**Background:**

Bovine congestive heart failure (BCHF) has become increasingly prevalent among feedlot cattle in the Western Great Plains of North America with up to 7% mortality in affected herds. BCHF is an untreatable complex condition involving pulmonary hypertension that culminates in right ventricular failure and death. Genes associated with BCHF in feedlot cattle have not been previously identified. Our aim was to search for genomic regions associated with this disease.

**Methods:**

A retrospective, matched case-control design with 102 clinical BCHF cases and their unaffected pen mates was used in a genome-wide association study. Paired nominal data from approximately 560,000 filtered single nucleotide polymorphisms (SNPs) were analyzed with McNemar’s test.

**Results:**

Two independent genomic regions were identified as having the most significant association with BCHF: the arrestin domain-containing protein 3 gene ( *ARRDC3*), and the nuclear factor IA gene ( *NFIA*, mid- *p*-values, 1x10 ^−8^ and 2x10 ^−7^, respectively). Animals with two copies of risk alleles at either gene were approximately eight-fold more likely to have BCHF than their matched pen mates with either one or zero risk alleles at both genes (CI _95_ = 3-17). Further, animals with two copies of risk alleles at both genes were 28-fold more likely to have BCHF than all others ( *p*-value = 1×10 ^−7^, CI _95_ = 4-206). A missense variant in *ARRDC3* (C182Y) represents a potential functional variant since the C182 codon is conserved among all other jawed vertebrate species observed. A two-SNP test with markers in both genes showed 29% of 273 BCHF cases had homozygous risk genotypes in both genes, compared to 2.5% in 198 similar unaffected feedlot cattle. This and other DNA tests may be useful for identifying feedlot animals with the highest risk for BCHF in the environments described here.

**Conclusions:**

Although pathogenic roles for variants in the *ARRDC3* and *NFIA* genes are unknown, their discovery facilitates classifying animals by genetic risk and allows cattle producers to make informed decisions for selective breeding and animal health management.

## Introduction

**Figure 1.**
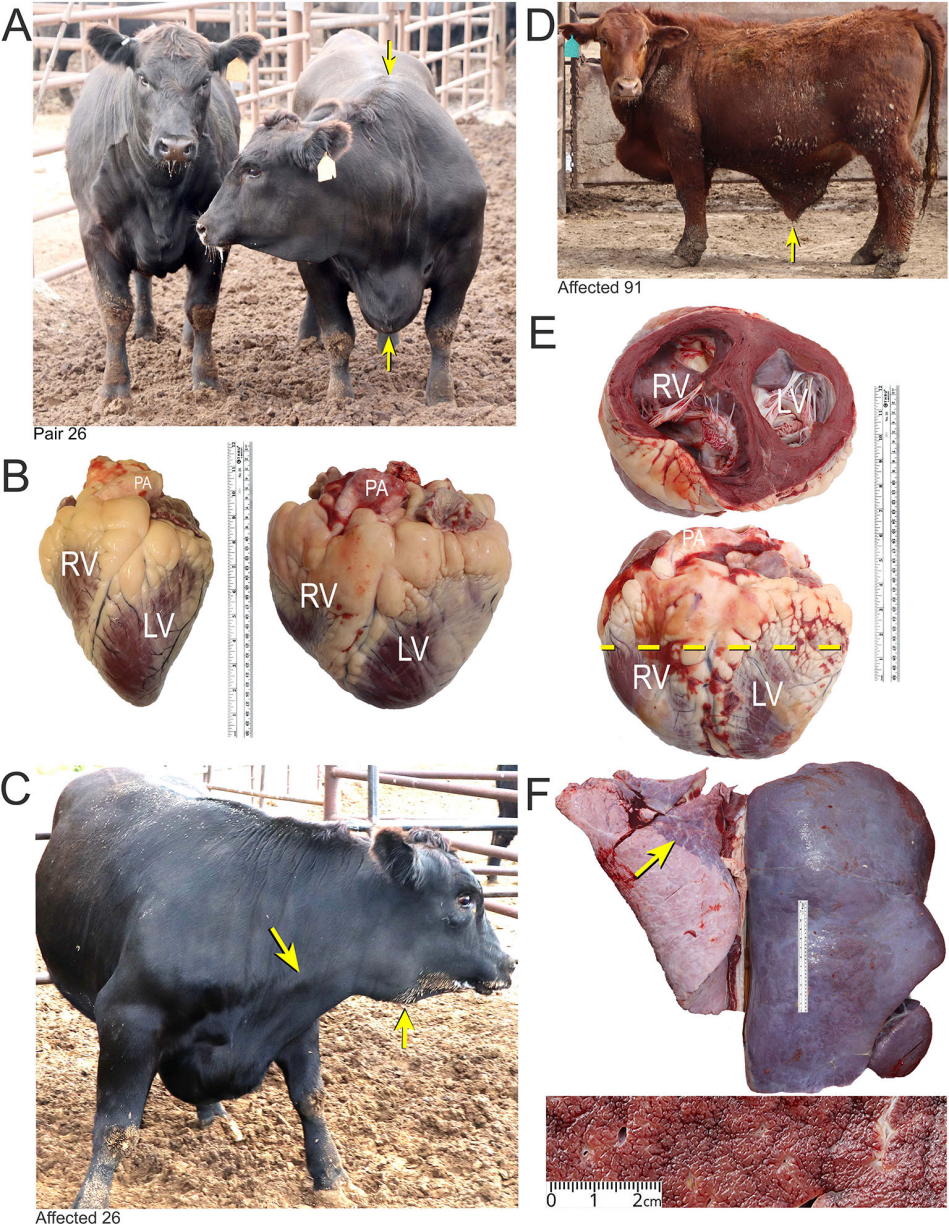
Features of BCHF in feedlot cattle. Panel A, matched pair number 26 with the clinical case (right) showing ventral edema and a drop in the withers (arrows). The latter occurs as the fluid accumulates in thorax pushing the shoulders outward. Panel B, the affected heart of case 26 (right) compared with a normal heart of a fattened American Angus heifer. Abbreviations: RV, right ventricle; LV, left ventricle; PA, pulmonary artery. Panel C, PA distension and mandibular edema in case 26 (arrows). Panel D and E, clinical case number 91 with ascites (arrow) and enlarged heart with dilated RV. Panel F, Enlarged, encapsulated liver of case 91 with “nutmeg liver” appearance in cross section due to hepatic venous congestion, and atelectasis in lungs (arrow).

Bovine congestive heart failure (BCHF) is a significant cause of death in feedlot cattle at the low to moderate elevations of the Western Great Plains of North America (800 to 1,600 m) ( Jensen *et al.*, 1976; Neary *et al.*, 2016). Feedlot cattle in this region are typically a mix of germplasm derived from popular British and Continental European breeds with more than 60% of the animals having a black hide from Angus contributions. Mortality from BCHF has reached 7.5% in severely affected pens of cattle, with annual losses exceeding $250,000 for a single operation ( Heaton *et al.*, 2019). Consequently, reducing the impact of BCHF is a priority for the U.S. cattle industry. The clinical features of BCHF in feedlot cattle are those caused by pulmonary hypertension, right heart failure, and passive liver congestion ( Figure 1). BCHF shares some similarities with the high-elevation “brisket disease” disorder that has been known in the Rocky Mountains of Colorado and Utah for more than 100 years, including severe ventral edema of the chest tissues ( Glover and Newsom, 1914, 1918). In the high-elevation disorder, the reduced partial pressure of oxygen causes pulmonary hypoxia, vascular resistance, arterial remodeling, and pulmonary hypertension. This condition has been attributed to “cor pulmonale” and eventually causes right ventricular overload and enlargement, ultimately leading to heart failure ( Hecht *et al.*, 1962). In BCHF, pulmonary arteries and arterioles also have lesions consistent with hypoxia-induced pulmonary hypertension ( Moxley *et al.*, 2019). However, it is unclear whether hypobaric hypoxia is the underlying cause. Some evidence suggests that left heart dysfunction may also play a role in initiating BCHF ( Krafsur *et al.*, 2019). Thus, disease pathogenesis of BCHF in feedlot cattle maintained at moderate elevations remains unclear.

Evidence in humans, mice, and cattle suggests that genetic factors influence the risk of pulmonary hypertension and right heart failure. In humans, dozens of inherited DNA sequence variants are known to directly or indirectly affect biological processes involved with cardiopulmonary disease ( Morrell *et al.*, 2019; Reza and Owens, 2020). In cattle, specific pathogenic mutations identified in the *PPP1R13L* and *OPA3* genes result in their loss of function (LOF) and cause recessive autosomal cardiomyopathies ( Simpson *et al.*, 2009; Owczarek-Lipska *et al.*, 2011). Human and mouse LOF mutations in *PPP1R13L* cause similar cardiomyopathies ( Falik-Zaccai, 2017; Herron *et al.*, 2005), as does mouse LOF mutations in OPA3 ( Davies *et al.*, 2008). These examples illustrate the fundamental conservation of mammalian genetics and diseases that can inform the search for optimal cardiovascular health for people and animals.

With regard to the BCHF disorder in Western Great Plains feedlots, there is only indirect evidence of an underlying genetic predisposition. Interviews with feedlot personnel at multiple affected operations indicated that cattle from different ranch sources varied markedly in their incidence of BCHF, despite being from the same breed and raised in the same general region with similar management conditions. Personnel also noted that in most years cattle from the same sources have a predictable prevalence and timing for developing BCHF in their feedlot environment (Heaton, M.P., 2017 unpublished). These observations are consistent with the hypothesis that genomic DNA sequence variants are influencing BCHF incidence, and each herd has its own frequency of the risk alleles due to founder effects. Based on comparative mammalian genetics, together with these anecdotal observations, we hypothesize that underlying genetic risk factors are influencing BCHF in feedlot cattle.

Candidate gene variants have not been confirmed as risk factors for BCHF in feedlot cattle. A first potential candidate gene variant was reported for cattle with high-altitude pulmonary hypertension ( Newman *et al.*, 2015). American Angus cattle affected with pulmonary hypertension at altitudes of 1,478 to 2,618 m had a higher frequency of the endothelial PAS domain-containing protein 1 gene ( *EPAS1*) encoding a hypoxia-inducible factor 2 alpha (HIF2α) missense variant. In addition, six other HIF2α polypeptide sequences were identified in cattle and used to evaluate their potential impact on the adaptive response to chronic hypoxia in U.S. cattle ( Heaton *et al.*, 2016). However, a retrospective study with 102 matched case-control pairs of feedlot cattle showed that none of the HIF2α isoforms encoded by *EPAS1* were associated with BCHF and indicated the need for a wider search ( Heaton *et al.*, 2019).

Here we used the same retrospective matched case-control design and the same 102 pairs of cattle from affected feedlots in the Western Great Plains (Table S1, *Extended data*, [Heaton *et al.*, 2019]), together with 778,000 single nucleotide polymorphisms (SNPs), to test the null hypothesis that significant association does not exist between haplotype tagging SNPs and BCHF. Our results rejected the null hypothesis and identified two distinct genomic regions harboring major genetic risk factors associated with BCHF. Cases were matched with contemporary pen mates to reduce confounding association signals that could be caused by population stratification and environmental differences. The strongest association signals were within the arrestin domain-containing protein 3 ( *ARRDC3*) gene on chromosome 7, and the gene for nuclear factor IA ( *NFIA*) on chromosome 3. The results have implications for deciphering underlying disease mechanisms, selection of breeding animals with reduced risk, and management of animals with high-genetic risk for BCHF.

## Methods

### Ethical statement

The experimental design and procedures used during this research project were reviewed and approved by the Institutional Animal Care and Use Committee of the University of Nebraska-Lincoln as previously described (Experimental Outline numbers 139 and 1172 [ Workman *et al.*, 2016; Heaton *et al.* 2019]). The animals were privately owned by commercial feeding operations, and the owners and management approved the use of animals for this study. In every instance, all efforts were undertaken to reduce animal suffering. In addition, animal welfare for these facilities was in accordance with the National Cattlemen’s Beef Association’s Beef Quality Assurance Feedyard Welfare Assessment program ( http://www.bqa.org/).

### Animals and study design

Paired samples from 102 affected feedlot cattle and their 102 unaffected matched pen mate controls were identified by monitoring approximately 140,000 total animals in four feedlots located in Nebraska and Wyoming at elevations ranging from 1,163 to 1,280 m. The samples were collected during a 16-month period spanning January 2017 to April 2018 and the 102 pairs originated from 30 different sources with the largest single source contributing 32 pairs ( Heaton *et al.*, 2019). The pen sizes ranged from 100 to 300 animals, and control animals were selected based on their willingness to be moved to the sampling area by riders on horseback. Candidate end-stage BCHF cases were identified by feedlot personnel who had experience with clinical signs of BCHF. Researchers were notified and initiated travel to the location (250 to 370 miles). With researchers present, the candidate case animal was euthanized by feedlot personnel and researchers performed necropsy.

The case definition requirement included two or more multiple clinical signs associated with BCHF including: ventral and intermandibular edema (‘brisket’ and ‘bottle jaw’), jugular vein distention and pulsation, ascites/abdominal swelling, and exophthalmia (‘bug-eye’)10,11. The case definition also included two or more non-specific clinical signs: dyspnea, abducted elbows, depression, drooped ears, intermittent watery orange diarrhea, tachycardia, exercise intolerance, open mouth breathing, and weight loss ( Heaton *et al.*, 2019). Cases were enrolled in the study only if there was a postmortem presumptive diagnosis of congestive heart failure at necropsy. Of the animals euthanized, the rejection rate for case enrollment was 20% (26/128). Unaffected pen mate controls were matched for source, arrival date, gender, and coat color. The case enrollment rate (102 per 140,000 feedlot cattle monitored, 0.073%) was negatively affected by: 1) removal of candidate case animals for emergency salvage slaughter, 2) removal of matched unaffected pen mates for beef processing, 3) the rapid onset of congestive heart failure and death, 4) reluctance of overburdened personnel to commit extra time despite the $500 indemnity offer for each matched pair identified early, and 5) exclusion of necropsied candidate animals meeting some, but not all, of the case definition criteria.

None of the 102 unaffected matched pen mate animals developed clinical BCHF signs prior to their processing into beef. Overall, the group of 204 calves was 93% solid black, 70% castrated males, and none had visible horns ( Heaton *et al.*, 2019). Most of the 204 calves were from known, well-managed herds that use American Angus genetics. However, breed information was not available for all animals. Tissue samples for genomic DNA isolation included V-shaped ear notches and EDTA whole blood. The ear tissue was desiccated with granular NaCl in the field and stored at -20°C upon return. The plasma and cellular fractions of EDTA whole blood were separated and frozen in liquid nitrogen *en route* and stored at -80°C upon return. Postmortem whole hearts used for gross morphological comparisons representing unaffected cattle were collected during federally-inspected beef processing at the USMARC abattoir from 21 purebred American Angus heifers raised and fattened at an elevation of 578 m.

A panel of 96 unrelated individuals from popular U.S. beef breeds (USMARC Beef Diversity Panel version 2.9 [MBCDPv2.9]) was used for genetic comparisons with case-control animals ( Heaton *et al.*, 2016). The panel design was based on a previous set of commercially-available U.S. sires from 19 breeds with minimal pedigree relationships ( Heaton *et al.*, 2001). Pedigrees were obtained from leading suppliers of U.S. beef cattle semen and analyzed to identify the least related individuals for inclusion. The number of sires representing each breed (four, five, or six) was based on their numbers of registered progeny circa 2000: Angus (n = 6), Hereford (n = 6), Charolais (n = 6), Simmental (n = 6), Red Angus (n = 6), Limousin (n = 6), Gelbvieh (n = 6), Brangus (n = 5), Beefmaster (n = 5), Salers (n = 5), Shorthorn (n = 5), Maine-Anjou (n = 5), Brahman (n = 5), Chianina (n = 4), Texas Longhorn (n = 4), Santa Gertrudis (n = 4), Braunvieh (n = 4), Corriente (n = 4), and Tarentaise (n = 4). On the basis of the number of registered progeny, the breeds were estimated to represent more than 99% of the germplasm used in the U.S. beef cattle industry. Based on pedigrees, the panel members contained more than 187 unshared haploid genomes (of 192 possible) and allowed a 95% probability of detecting any allele with a frequency greater than 0.016.

### Genotype scoring, SNP filtering, and population substructure evaluation

Unless otherwise indicated, reagents were molecular-biology grade. DNA from ear notches was extracted by standard phenol/chloroform procedures, dissolved in a solution of 10 mM TrisCl, 1 mM EDTA (TE, pH 8.0) and stored at 4°C. ( Heaton *et al.*, 2008). SNP genotypes for variants on the BovineHD BeadChip array were scored at GeneSeek Inc. (Lincoln, NE, USA), according to manufacturer’s instructions (Illumina, Inc., San Diego, CA, USA). The unfiltered map and ped files from the 102 BCHF case-control animals (Files S1 and S2, *Extended data*) were analyzed for association with BCHF with PLINK v1.90b6.5 64-bit (13 September 2018) software ( Chang *et al.*, 2015). The pairs (1 to 102) were designated in the PLINK family ID field. SNPs were removed for having a minor allele frequency less than 0.05 (--maf option). This cutoff was conservative since the frequency of the affected individuals was set at 0.50 *a priori* in this matched case-control design. The missing genotype rate threshold was set at 0.05 (--geno option) and the Hardy-Weinberg equilibrium test with the mid- *p* adjustment threshold was set to 0.0001 (--hwe midp option) ( Graffelman and Moreno, 2013). These filters were applied for 204 samples of which 142 were listed as males (steers) and 62 were listed as females (heifers). The animal genders were tested with the --check-sex option and a control animal was identified as being misclassified as a male, leaving 141 steers and 63 heifers, and pair 21 mismatched for sex with the case being a steer and the control being a heifer (Files S3 and S4, *Extended data*).

Since unrecognized population stratification among individuals may obscure association signals and lead to incorrect findings ( Deng, 2001; Cardon and Palmer, 2003), multidimensional scaling (MDS) was used with a matrix of pairwise identity-by-state (IBS) distance to visually evaluate the similarity between individuals and pairs. Using PLINK software, unfiltered input map and ped files from the 102 BCHF case-control animals (Files S1 and S2, *Extended data*) were merged with likewise unfiltered input map and ped files from the USMARC Beef Cattle Diversity Panel version 2.9 (MBCDP v2.9) genotyped with BovineHD BeadChip array ( Heaton *et al.*, 2016) (Files S5 and S6, *Extended data*). Once merged, the data was filtered as described above to produce a new map and ped files (Files S7 and S8, *Extended data*). MDS data was produced with PLINK software v1.9 and plotted with Microsoft Office 365 Excel software. The IBS/IBD computation was run on the filtered SNP sets with the --genome option. The computation excluded 12,591 variants on non-autosomes from the IBD calculation. Plotting the --cluster output values in the first and second dimensions (i.e., C1 values against C2) gave a scatter plot in which each point is an individual; the two axes correspond to a reduced representation of the data in two dimensions, which facilitates cluster identification. Standard classical (metric) multidimensional scaling was used.

### McNemar’s test with SNP genotypes

The McNemar’s test is a statistical test for paired nominal data ( McNemar, 1947). Unlike a traditional case-control GWAS where the scope of inference is over two groups of individuals, a McNemar’s test analysis for a case-control GWAS uses only the two matched individuals as the unit of comparison. This approach previously showed significant power to detect the association of *TMEM154* missense variants with ovine progressive pneumonia with 69 matched pairs and a 50k bead array ( Heaton *et al*., 2012). In addition, McNemar’s test analysis is not formally available as a convenient option in commonly used, open-source GWAS toolsets that can analyze 560,000 SNPs such as PLINK (v1.90b6.5). The Cochran-Mantel-Haenszel (CMH) test ( Mantel and Haenszel, 1959) is available in PLINK and can be run as a specialized case with two clusters; however, it has limited options and does not perform essential McNemar computation with raw data. Thus, a custom workflow was developed with a programming and numeric computing platform (MatLab v.2021.2, MathWorks, Inc., Natick, MA) to produce the critical tables of binomial outputs and McNemar statistics for the filtered SNP genotypes.

In McNemar’s test, informative pairs are those which contain exactly one animal with the hypothetical risk allele genotype, i.e., Table 1, quadrants *b* and *c.* Depending upon the scenario tested, the risk allele genotype may include one or two copies of the variant. The ratio of the discordant pairs ( *b/c*) provides evidence to reject the null hypothesis. At each biallelic SNP locus, alleles were tested separately in three scenarios as a potential disease risk factor: 1-copy, 1- or 2-copies, and 2-copies, producing six sets of McNemar’s test statistics. The presence of a potential risk allele(s) was scored for both animals in every matched pair and used to populate the McNemar’s 2 × 2 contingency table for a given SNP. The presence (+) or absence (-) of a possible genetic risk factor was assigned to each animal and the matched pair was then classified by one of four possible binomial outcomes ( Table 1, quadrants). A genetic “risk” allele was indicated in the 102 matched pairs when quadrant *b* was greater than quadrant *c* (i.e., odds ratio (OR) > 0). Conversely, a “protective” allele was indicated when quadrant *c* was greater than quadrant *b* (i.e., OR < 0).

**Table 1.** The four binomial outcomes possible in this McNemar’s test ^a^.

	Control has risk allele(s)	Control lacks risk allele(s)	Row totals
Case has risk allele(s)	( **+**, **+**) *a*	( **+**, **-**) *b*	*a* + *b*
Case lacks risk allele(s)	( **-**, **+**) *c*	( **-**, **-**) *d*	*c* + *d*
	*a* + *c*	*b* + *d*	*n*

^a^ Each binomial outcome is a case-control pair, i.e., (case, control) with the sign indicating the presence or absence of the risk allele. For example, (+,-) indicates an informative outcome where the case has the risk allele and the control does not. The uninformative pairs are (+,+) and (-,-) where each member of the pair has identical risk allele status. Note that this table can be set up for scenarios of 1, 1 or 2, and 2 copies of risk alleles.

McNemar’s test analysis for a GWAS imposes statistical constraints at the SNP level that are not present in conventional GWAS. For example, the McNemar’s test *p*-values for rejection of the null hypothesis were computed with the binomial distribution. This is different from a conventional retrospective case-control GWAS, where the theoretical distribution of *p*-values may be modeled with Fisher’s exact test statistics (or derivative). The binomial distribution in the McNemar’s analysis generated discrete *p*-values (e.g., exact *p* and mid *p*) at each SNP site that were dependent on the discordant pairs observed (i.e., *b* + *c* and *b*/ *c*) for each inheritance model (e.g., 1-copy, 1- or 2-copies, and 2-copies). Thus, *p*-values were computed *de novo* at each locus. To the best of our knowledge, the theoretical genome-wide distribution of McNemar’s *p*-values at the SNP level has not been previously described in the GWAS context. Briefly, the input files consisted of PLINK map and ped files with the ped file modified to include the pair identifier in the first column, and the rows sorted in descending pair order from 1 to 102 with the unaffected animal listed first in the pair. The case and control phenotype were coded in the sixth column, with the unaffected control animal coded with 1 (PHENO = 1) and the affected case animal with a 2 (PHENO = 2). The analysis evaluated each SNP for possible risk alleles in the context of McNemar’s test as described in README.md file in the GitHub code repository McNemarsSNPAnalysis. Alternatively, the analysis pipeline can be run online within a copy of the McNemar’s SNP Analysis Compute Capsule at CodeOcean. The PLINK map and ped files contained 141 steers and 63 heifers, and SNPs assigned to chromosome 0 through 32 (i.e., included unassigned SNPs, chromosome X, Y, and mitochondrial DNA; Files S9 and S10 *, Extended data*). In the first stage of the workflow, biallelic SNP heterozygous animal genotypes were converted to a one-letter International Union of Pure and Applied Chemistry and International Union of Biochemistry and Molecular Biology (IUPAC/IUBMB) ambiguity codes used for nucleotides (i.e., R = a/g, Y = c/t, M = a/c, K = g/t, S = c/g, W = a/t (‘Nomenclature Committee for the International Union of Biochemistry [NC-IUB]. Nomenclature for incompletely specified bases in nucleic acid sequences. Recommendations 1984’, 1986) and homozygous animal genotypes were converted to a one-letter code of the same letter (i.e., A, C, T, G).

The McNemar mid- *p* test was used, since it is an improvement over the exact conditional test ( Fagerland *et al.*, 2013). Briefly, the McNemar mid- *p*-value was calculated with equation 1:

$$2*\left[\text{binomcdf}\left(n_{12},b+c,0.5\right)-½\;\text{binompdf}\left(n_{12},b+c,0.5\right)\right]$$ (1)

where binomcdf = binomial cumulative distribution function, binompdf = binomial probability distribution function, *b* = the number of informative pairs with only the case having the risk factor (“successes”), *c* = the number of informative pairs with only the control having the risk factor (“failures”), *b* + *c* = “trials”(i.e., the number of discordant pairs), n _12_ = the smaller value of *b* or *c*, and 0.5 = “probability of success” (i.e., probability of being *b* or *c* by chance). In Excel, this formula returns the McNemar mid- *p*:

$$=2*(\left(\text{BINOM}.\text{DIST}\left(\left(\text{IF}\left(b<c,b,c\right)\right),b+c,0.5,\text{TRUE}\right)\right)-\left(½\left(\text{BINOM}.\text{DIST}\left(\left(\text{IF}\left(b<c,b,c\right)\right),b+c,0.5,\text{FALSE}\right)\right)\right)$$

where “TRUE” was the option for the cumulative distribution function and “FALSE” was the option for the probability distribution function.

The quantile-quantile (Q-Q) plots of the distribution of the test statistics are challenging to produce because McNemar’s test data are discrete for a given number of total pairs and the fraction of discordant pairs (( *b* + *c*)/n, Table 1). This requires the computed theoretical expected distribution of *p*-values to mirror the dependency of the observed distribution of *p*-values on the fraction of discordant pairs. Thus, for a given informative pair ( *b* + *c*) value, theoretical *p*-values in Q-Q plots were computed by multiplying the number of observed *p*-values by the predicted normalized binomial distribution of all possible *b* and *c* values, as defined by the *p*-value metric used, e.g. exact *p* or mid *p.* The fraction of discordant pairs is also influenced by minor allele frequency, as the opportunity for the observation of discordant pairs diminishes with frequency.

Systematic use of *q*-values in genome-wide tests provides a balance between the number of true and false positives while being automatically calibrated and readily interpreted ( Storey and Tibshirani, 2003). The *q*-value of a genome-wide data set is the expected proportion of false positives incurred ( Storey and Tibshirani, 2003). For instance, a *q*-value threshold of 5% results in the false discovery rate (FDR) of 5% of the significant features being truly null. The mafdr function in the Matlab Bioinformatics Toolbox (version 2021.2) was used to compute the *q*-values from input *p*-values according to procedures previously described ( Storey, 2002). The tuning parameter, λ, was used to estimate the *a priori* probability that the null hypothesis is true ( equation 2):

$$(\pi_{0}\equiv \frac{\text{number of true null observations}}{\text{number of}\;\mathrm{all}\;\text{observations}}).$$ (2)

This parameter was computed both with the ‘bootstrap’ and ‘polynomial’ methods in the mafdr function yielding similar results. The polynomial method was a cubic polynomial fit of λ to $\hat{\pi_{0}}\;\left(\lambda \right)$ (equation 6 in [ Storey, 2002]), as demonstrated for the 2-copy risk allele model (Figure S1A, *Extended data*). In contrast to previous assumptions where the frequency of *p*-values tend to remain constant with *p*-values > 0.5 ( Storey, 2002), *p*-values in the present report exhibit an increasing frequency > 0.4 and are not uniformly distributed because of the discrete results generated by the McNemar’s test (Figure S1, *Extended data*). Consequently, the polynomial method of computing $\hat{\pi_{0}}$ $\left(\lambda \right)$ was negatively affected, resulting in poor cubic polynomial fit with R ^2^ = 0.17, especially as λ approaches 1. Recalling that when the *p*-value equals one, McNemar quadrants *b* and *c* are also equal, and thus significant SNP associations are not expected; stated another way, it would be extremely unusual for any SNP with a McNemar *p*-value of one to be truly associated with disease risk. The computed $\hat{\pi_{0}}$ value tends to increase as the frequency of discrete *p*-values inflate. This in turn decreases the number of observations that are found to be non-null significant SNPs that pass a given *q*-value threshold. For instance, in the scenario where homozygous major alleles are evaluated as genetic risk factors, the number of SNPs passing the *q*-value = 0.05 threshold using the polynomial method was 55 with a $\hat{\pi_{0}}$ = 0.8486 while the bootstrap methods found 73 significant SNP with a $\hat{\pi_{0}}$ = 0.5709. Therefore, for a given *q*-value threshold, the computed SNPs found to be significant in this McNemar analysis were conservative compared to the more conventional GWAS with continuous *p*-values distributions that tend to “flatten” with *p*-values > 0.5, reducing $\hat{\pi_{0}}$ and increasing the number of non-null significant SNPs found using the canonical Storey analysis as embodied in the mafdr function. To explore the expected genome-wide *p*-values for a McNemar-based Q-Q plot, the observed normalized *p*-value distribution (parametrically dependent upon McNemar’s quadrants *b* and *c* [informative pairs] for a given *b* + *c*) was compared to the normalized expected theoretical distribution of *p*-values using the same parameters (i.e., observed *b* + *c* values) at each SNP. For example, computing the Q-Q plot’s expected distribution of *p*-values over all SNP loci required that the expected *p*-value be computed relative to the number of observed informative pairs at each locus. In addition, it was necessary to confirm that the approximations used for computing the false discovery rate to correct for multiple test biases were still valid for discrete *p*-values.

The six sets of McNemar’s pair classifications and test statistics were tallied for each SNP allele and exported to a text file for importation into spreadsheet software (Excel Version 1902, Build 11328.20420). A spreadsheet environment allowed for the SNP set to be used in calculations for McNemar’s statistics such as odds ratios (OR), *p-*values, and McNemar Chi-squared statistics. After McNemar’s testing, SNPs were removed due to their assignment to chromosomes 0, 31, and 32. These SNPs represent markers on unmapped chromosomes, SNPs on chromosome Y, and mitochondrial SNPs. SNPs were also removed if there were no informative pairs resulting from their pairwise analysis (i.e., McNemar quadrants *b* + *c* = 0).

The upper and lower CI _95_ for the OR were calculated in equations 3 and 4:

$$e^{\left(\ln,\left(\text{OR}\right),+,1.96,\left(\mathrm{SE}\;\text{of}\;\ln\left(\text{OR}\right)\right)\right)}$$ (3)

$$e^{\left(\ln,\left(\text{OR}\right),-,1.96,\left(\mathrm{SE}\;\text{of}\;\ln\left(\text{OR}\right)\right)\right)}$$ (4)

respectively, where e = the natural base, ln = natural logarithm, and SE = the standard error ( $\sqrt{1/b+1/c}$ ) . The McNemar test statistic $\chi^{2}$ = ( *b*- *c*) ^2^/( *b*+ *c*), and $\chi^{2}$ with the continuity correction = (| *b* - *c*|- 1) ^2^/( *b* + *c*). The effect size (Cohen’s g) was the larger of ( *b*/( *b*+ *c*) or *c*/( *b*+ *c*)) - 0.5 (Cohen 1988, Statistical Power Analysis for the Behavioral Sciences).

The spreadsheet format allowed for custom labeling of column headers, row sorting, and color-coded heat mapping. For example, it was useful to sort each of the six sets of data by their best McNemar’s *p*-value in descending order and color code the rows in blocks of 100 (e.g. purple, red, yellow, dark green, light green). This helped visually identify the top 500 best markers in each data set. When resorted by chromosome and position, the *p*-value color-coded rows produced an obvious visual symmetrical pattern due to the biallelic nature of SNPs and the dichotomous phenotype. An exception to the pattern occurred in regions where the major and minor allele frequency were equivalent since their assignment was arbitrary for the set of 204 animals.

The spreadsheet format also allowed for manual production of Manhattan plots from McNemar’s data for three inheritance models of the two alleles for each SNP. Briefly, the ARS-UCD1.2 reference assembly SNP coordinates from the PLINK map file (i.e., chr and position) were converted to a linear genome position for the x-axis of the Manhattan plot so that every SNP had a new relative coordinate on a whole genome scale from 1 to 2,651,083,094 bp. This was accomplished by adding the position value of the most terminal SNP on chromosome 1 to each of the SNP position values on chromosome two. This was repeated for each consecutive chromosome (1 to 29 and X). The Bonferroni correction for multiple testing was used at the 0.05 significance level (α) as a conservative genome-wide estimate of significance ( Johnson *et al.*, 2010) ( Equation 5):

$$\text{Bonferroni genome}-\text{wide significance threshold}=\alpha /\left(\text{filtered SNPs}\right)$$ (5)

### Power analysis

Sample size power analysis for the McNemar’s test was performed with PASS 2019 Power Analysis and Sample Size Software, version 19.0.2 (NCSS, LLC., Kaysville, Utah, USA). The sample size of 102 matched pairs achieved 95% power to detect an OR of 8.51 and 2.94 with a two-sided McNemar’s test and a corrected significance level of 0.01 (α) within the range of 25% and 70% discordant pairs, respectively. Power is the probability of rejecting a false null hypothesis, while α is the probability of rejecting a true null hypothesis.

### CMH test

McNemar’s test analysis was compared to that obtained with the CMH association statistic ( Mantel and Haenszel, 1959) in PLINK v1.90b6.5. The 102 matched pairing assignments were coded in the fam file in the third column to identify 102 “clusters” (e.g., Pair _1_, Pair _2_, Pair _3_, …Pair _102_) for use with the CMH association statistic. The --adjust option was also used to produce a sorted list of CMH association results. The Q-Q plot of the distribution of the test statistics was produced with the qqman R package on CRAN: (Turner, SD. 2014. bioRxiv) with 64-bit R version 3.5.1 (2018-07-02).

### Testing BCHF cases and controls for bovine viral diarrhea virus (BVDV) infection

Blood samples from BCHF cases and controls were tested for infection with BVDV, a possible confounding disease condition of feedlot cattle that is easily detected. However, since these animals were not serially sampled, a positive test result would not distinguish between acute and persistent infection. Blood samples for the first 13 case-control pairs were collected as EDTA whole blood, stored at -80 ^o^C, and used for BVDV testing. Blood samples for the remaining 89 case-control pairs were processed by first separating the plasma by centrifugation and storing it separately at -80 ^o^C prior to its use in BVDV testing. EDTA blood or plasma was pooled into groups of five or 10, respectively, and total RNA was extracted with a mono-phasic solution of phenol and guanidinium thiocyanate (TRIzol LS, Invitrogen-Thermo Fisher Scientific, Waltham, MA, USA) according to the manufacturer’s specifications. BVDV genomic RNA was detected with a previously published, real-time assay that used quantitative reverse transcription polymerase chain reaction (RT-qPCR) primers (5′-GGGNAGTCGTCARTGGTTCG-3′; 5′-GTGCCATGTACAGCAGAGWTTTT-3′) and probe (5′-6-FAM-CCAYGTGGACGAGGGCAYGC-BHQ1-3′) ( Mahlum *et al.*, 2002). The assay reagents were purchased as a commercial kit (Qiagen OneStep RT-PCR, Germantown, MD) and used according to the manufacturer’s instructions. Briefly, cyclic amplification was conducted in a 25 μL reaction containing 5 μL of extracted RNA, 4.5 mM magnesium chloride, 400 μM dNTP, 0.4 μM of each forward and reverse primer, 0.2 μM probe, and 1 μL of an enzyme mix containing reverse tran-scriptase and a “hot-start” *Taq* polymerase. Cycling conditions included reverse transcription at 50°C for 30 minutes, inactivation of the reverse transcriptase enzyme and activation of the *Taq* polymerase at 95°C for 15 minutes, and 40 cycles of 94°C for 30 seconds, 55°C for 60 seconds, and 72°C for 60 seconds. Positive, negative, no-template, and extraction control samples were included in each group of samples tested.

### SNP genotype assays for *ARRDC3* and *NFIA*

Custom SNP genotype tests were developed on a commercially available genotyping platform that uses “padlock” oligonucleotide probes, combined with fluorescently-labeled probes and isothermal rolling circle amplification technology, for the qualitative detection of alleles (MatMaCorp, Lincoln, NE, USA). Padlock probes are long oligonucleotides whose ends are complementary to adjacent target sequences. The primary target SNPs included *ARRDC3* C182Y (ARS-UCD1.2 chr7:90845941, rs109901274, BovineHD0700027239, BCHF5) and *NFIA* intron 4 (ARS-UCD1.2 chr3:84578325, rs136210820, BovineHD0300024307, BCHF31). However, genotyping tests involving tightly linked SNPs were also used on occasion. The assay steps consisted of sample processing (blood and ear notch), target amplification, and detection. Samples were processed and analyzed on the Solas 8 instrument per the manufacturer’s instructions. Briefly, samples were mixed with a proprietary lysis buffer and heated for three minutes at 95°C. Kit reagents were added to perform an initial PCR amplification with padlock probes. The machine assay conditions were SNP-specific and uploaded to the machine via the internet by the supplier: an initial heating phase at 93° for 3 min, followed by 30 cycles at 93° for 20 s and 57°C for 30 s. Detection of the ligated circular oligonucleotide products in the Solas 8 instrument was accomplished with fluorescently labeled probes and isothermal rolling circle amplification. The total reaction time was approximately two hours, and the genotype assignments were made in real time by the instrument, and the real-time graphical outputs were visually reviewed for quality assurance. Assay performance was validated with known genotypes derived from whole genome sequencing and the BovineHD BeadChip array from a beef cattle diversity panel ( Heaton *et al.*, 2016). Ambiguous genotype results from animals of unknown genotypes were resolved by repeat testing with a second assay designed for the opposite strand of *ARRDC3* C182Y (ARS-UCD1.2 chr7:90845941, rs109901274, BovineHD0700027239, BCHF5r) and the *NFIA* intron 4 SNP (ARS-UCD1.2 chr3:84580655, rs133192205, BovineHD0300024308, BCHF32).

### Protein folding prediction of ARRDC3 structures

The predicted protein structure of the bovine ARRDC3 was compared with the that from humans. These two proteins were 99% identical across all 414 amino acids (The National Center for Biotechnology Information [NCBI] reference sequences NP_001069725.1 and NP_065852.1, respectively). An artificial general intelligence software that uses machine learning was used to fold the ARRDC3 proteins (ColabFold v1.5.5: AlphaFold2 using MMseqs2; ( Mirdita *et al.*, 2022). Each of the three ARRDC3 sequences was folded separately, and the .pdb structure file with the highest confidence was selected as the best representation of the biologically relevant structure. The two ARRDC3 variant files were loaded into a molecular graphics system (PyMOL Molecular Graphics System v2.5.3; Schrödinger, LLC, New York, NY), aligned, and rotated to view the variant region.

## Results

### Genotype scoring, SNP filtering, and population substructure

**Figure 2.**
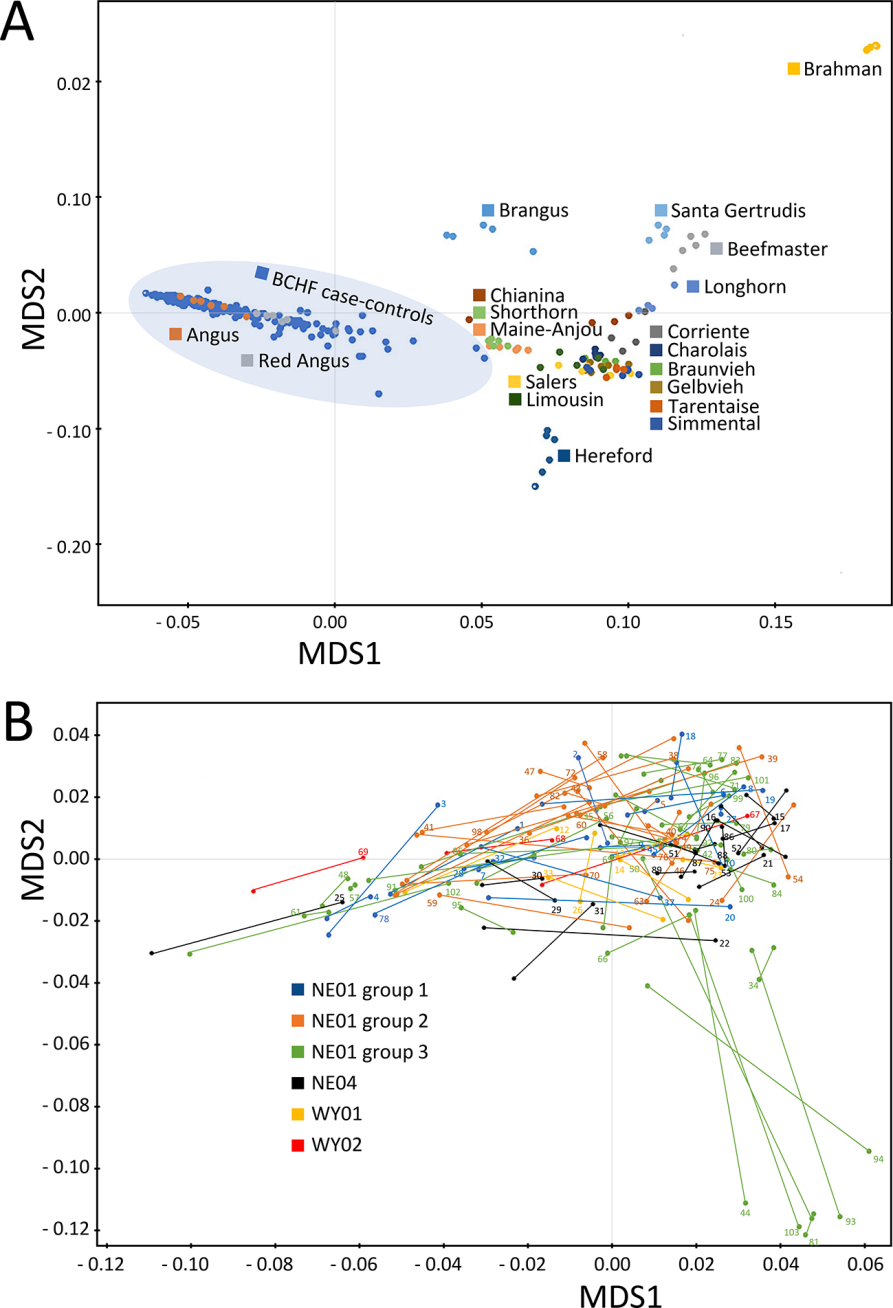
MDS plot with pairwise IBS distances between breeds and paired individuals. Panel A, genetic distances between BCHF case-control animals and diverse purebred bulls from 19 U.S. beef breeds. Panel B, genetic distances between matched pair from different feedlot groups and locations in Nebraska (NE) and Wyoming (WY). Lines connecting dots identify each matched pair with numbers corresponding to the pair ID (Table S1) and the clinical case.

Approximately 778,000 SNPs were scored for 204 animals in matched case-control pairs with a total genotyping rate of 0.99 (Files S1 and S2, *Extended data*). Subsequent filtering removed 216,000 SNPs with either low minor allele frequencies, missing genotypes, or deviation from Hardy-Weinberg expectations (173,000, 42,000, and 1,000, respectively). Approximately 560,000 remaining SNPs (Files S3 and S4, *Extended data*) were used for MDS analysis of pairwise IBS distances of the 204 animals from the 102 matched pairs, together with a 19-breed beef diversity panel. The feedlot animals from the 102 matched pairs were tightly clustered with purebred American Angus and Red Angus cattle ( Figure 2A). A higher resolution plot with the 102 pairs of BCHF cases and matched controls showed there were seven outliers from one feedlot group, of which five were cases and two were controls ( Figure 2B, lower right quadrant). However, many of the matched pairs had short IBS distances, indicating close genetic relationships between random penmates, as would be expected from animals originating from the same ranch. Based on the relatively low number of outliers that were BCHF cases, and the distances of their matched pairs, corrections for population stratification were not made in subsequent analyses.

### Genome-wide McNemar association testing

**Figure 3.**
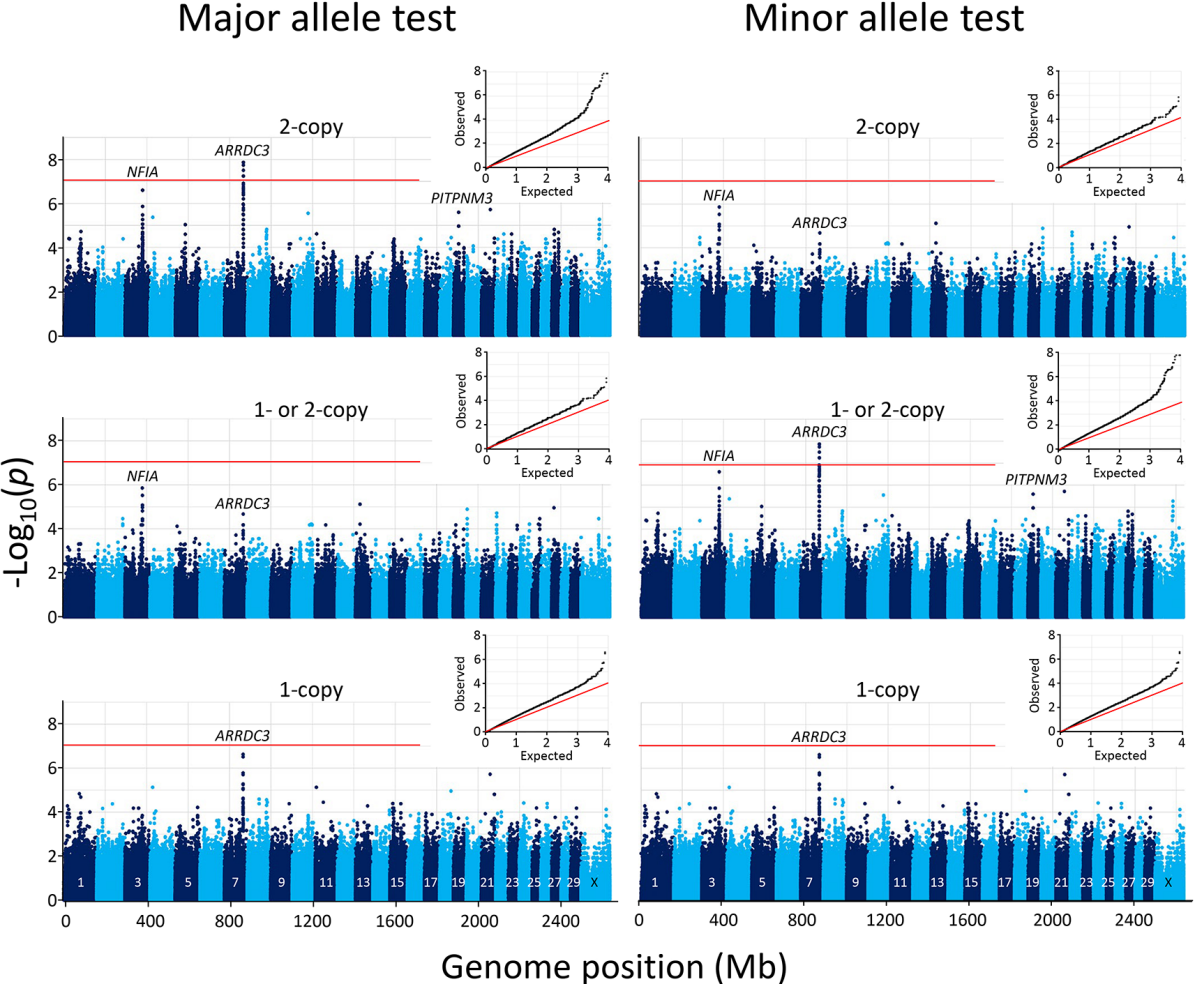
Manhattan and Q-Q plots of genome-wide association with 102 BCHF matched case-control pairs. The mid- *p*-values from the McNemar’s association test were used with major (left) and minor alleles (right). In both panels, the inset shows the Q-Q plot of the distribution of the test statistics and the red horizontal line shows the Bonferroni correction at an α of 0.05.

The CMH test was first used to analyze the data set with PLINK software, since it was available and tests for SNP-disease association conditional on clustering of the individuals and is a generalization of McNemar’s test. The CMH test is expected to give the same results as McNemar’s test when each cluster contains a pair of individuals since their test statistics are identical when each stratum shows a pair. However, CMH output in PLINK did not provide easy access to the individual McNemar’s quadrants results for each SNP nor the ability to test 1-copy, 1 or 2-copy, and 2-copy risk allele models. Thus, we developed a custom process on a programming and numeric computing platform as described in the Methods section. After producing the tables of McNemar test statistics (File S11, *Extended data*), SNPs were further removed because they had no informative pairs (n = 704), were unmapped (n = 668), on the chromosome Y (n = 31), or on the mitochondrial genome (n = 1) (File S12, *Extended data*). A Manhattan plot of the resulting SNPs revealed that the most significant genome-wide associations were clustered on chromosome 7 at the *ARRDC3* gene ( Figure 3). No other genomic region contained SNPs that met the Bonferroni threshold at the 0.05 genome-wide significance level (-log _10_ = 7.05).

**Figure 4.**
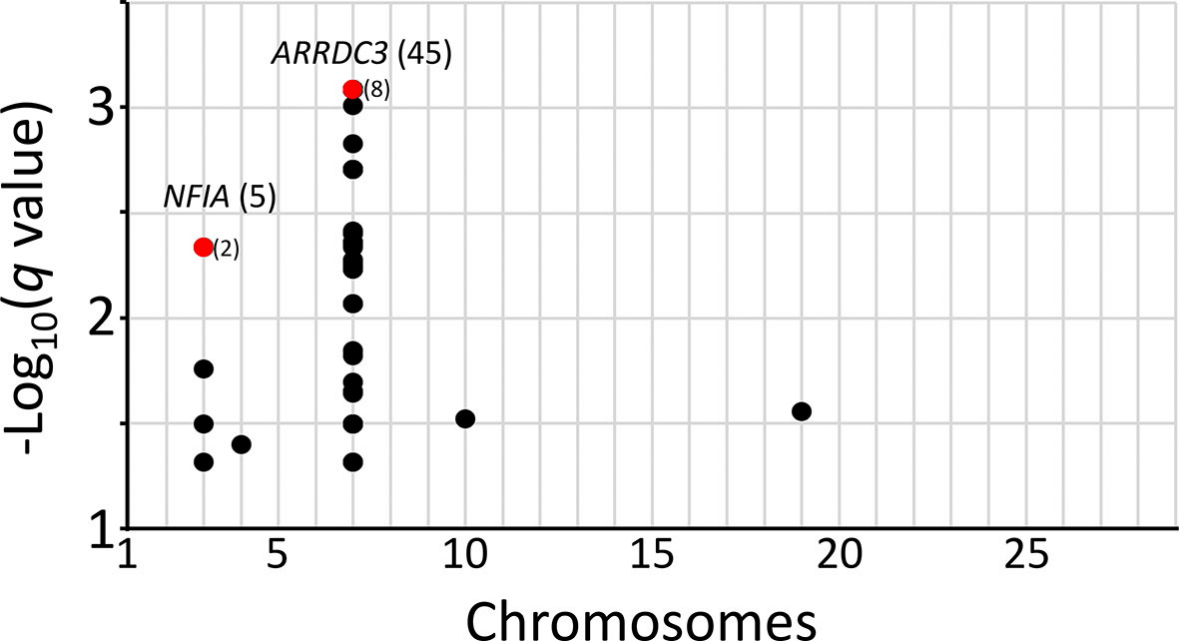
Genome-wide SNPs with a *q*-value threshold less than 0.05 FDR. The *q-*values for SNPs were evaluated for the two-copy risk allele model and plotted by chromosome. Numbers in parentheses are linked SNPs with *q*-values less than 0.05. Red dots and parentheses represent multiple linked SNPs with equivalent *q*-values.

While the Bonferroni correction protects against Type I errors (false positives), it is vulnerable to Type II errors (false negatives). To strike a balance between the number of true and false positives, *q*-values were calculated for the McNemar data set to identify regions surpassing a 5% FDR. The *q*-value threshold is the expected proportion of significant SNP associations that are false leads. Only models that compared homozygous risk alleles against all other genotypes yielded significant results. There were 53 genome-wide autosomal SNPs passing the 5% FDR: 49 were associated with *ARRDC3* on chromosome 7, five were associated with *NFIA* on chromosome 3, and one SNP each was associated with a region on chromosomes 4, 10, and 19 ( Figure 4, File S13 *Extended data*). Since the three SNPs on chromosomes 4, 10, and 19 correspond to the expected proportion of false leads, these regions were dismissed for the purposes of the present study. The remaining genomic regions associated with *ARRDC3* and *NFIA* genes were further characterized.

**Figure 5.**
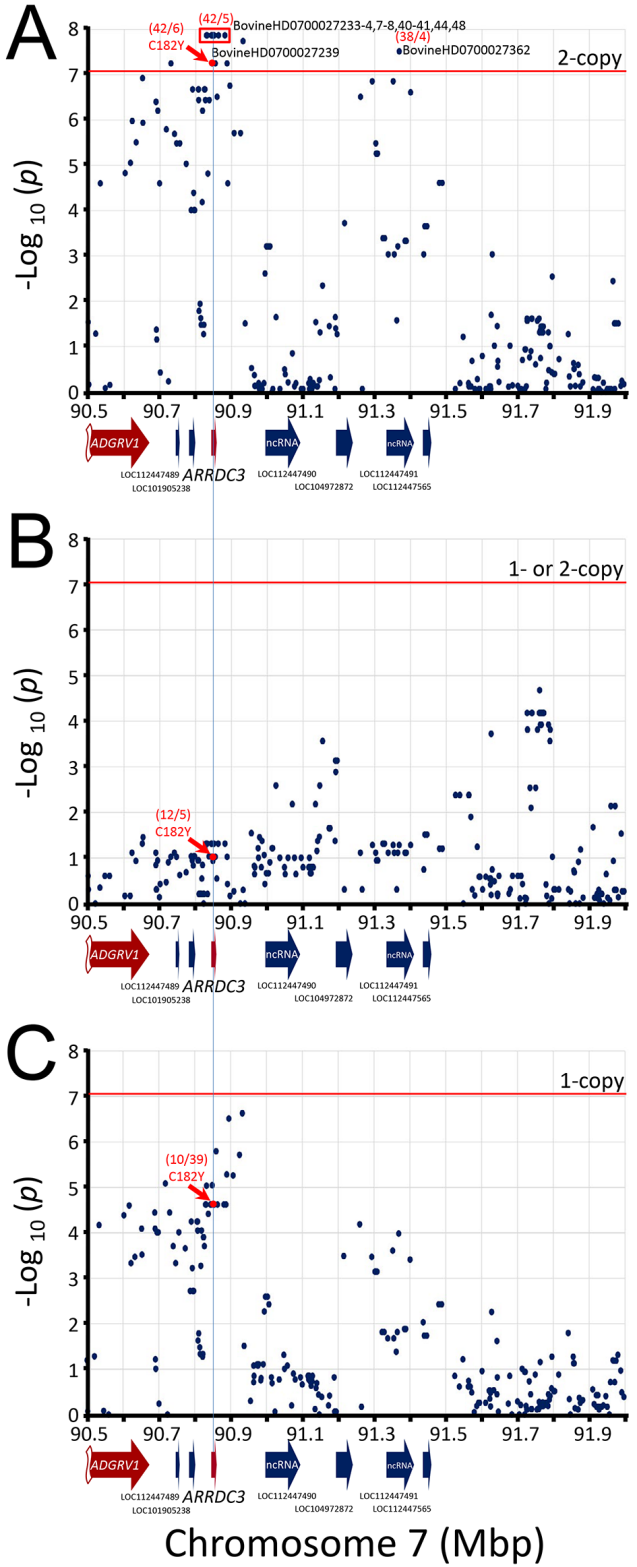
Manhattan plot of *ARRDC3* gene region. The mid- *p*-values from the McNemar’s association test of the most frequent SNP alleles were used. Panel A, test requiring the presence of homozygous risk alleles. Panel B, test requiring the presence of either 1- or 2-copies of the risk alleles. Panel C, test requiring heterozygosity for the risk alleles. The red horizontal line shows the Bonferroni correction at the 0.05 significance level. The red numbers in parentheses are McNemar's informative pairs from quadrants *b* and *c* (i.e., “ *b*/ *c*“, Table 1). The ratio of informative pairs indicates the strength of the association, and whether the allele is positively or negatively associated with BCHF (i.e., *b/c* > 0 or < 0).

**Figure 6.**
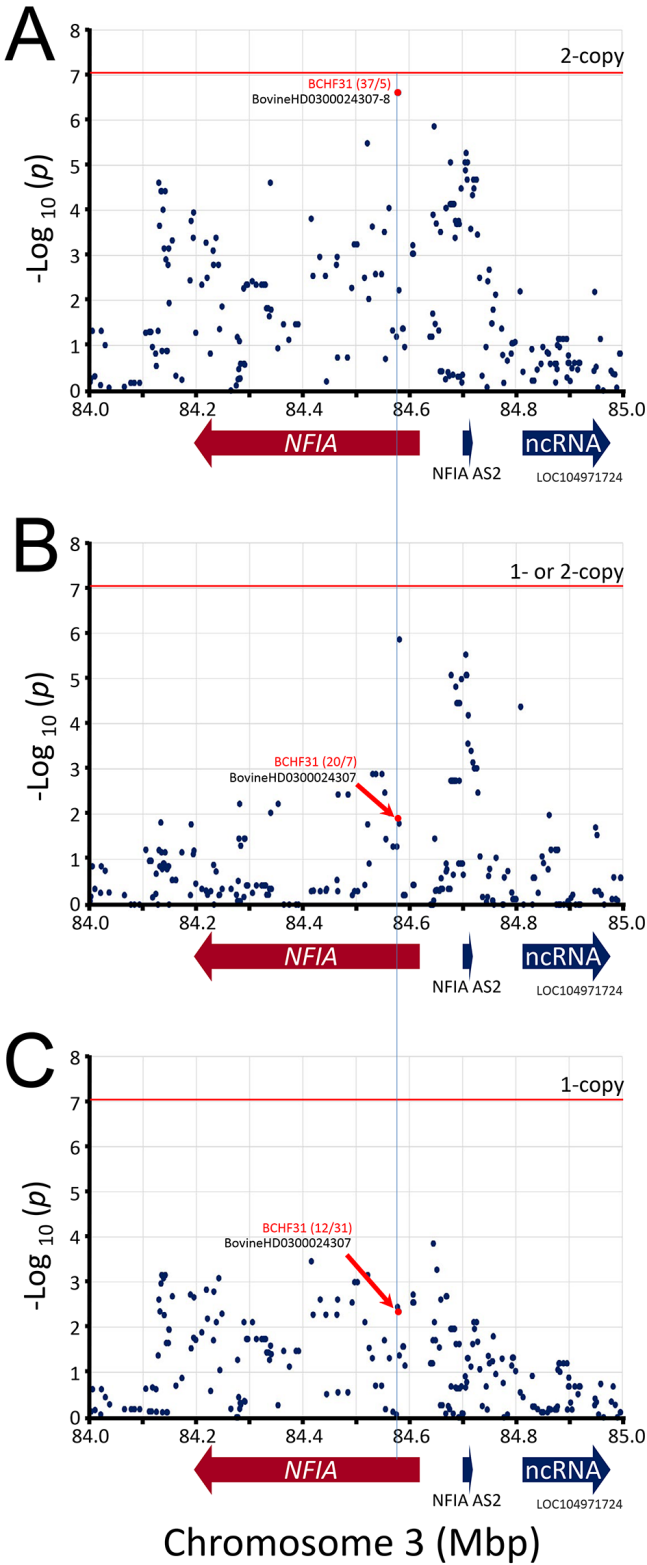
Manhattan plot of *NFIA* gene region. The mid- *p*-values from the McNemar’s association test of the most frequent SNP alleles were used **.** Panel A, test requiring the presence of homozygous risk alleles. Panel B, test requiring the presence of either 1- or 2-copies of the risk alleles. Panel C, test requiring heterozygosity for the risk alleles. The red horizontal line shows the Bonferroni correction at the 0.05 significance level. The red numbers in parentheses are McNemar's informative pairs from quadrants *b* and *c* (i.e., “ *b*/ *c*“, Table 1). The ratio of informative pairs indicates the strength of the association, and whether the allele is positively or negatively associated with BCHF (i.e., *b/c* > 0 or < 0).

The best associated region spanned 52 kb and was roughly centered on the *ARRDC3* gene. This region contained eight equivalent linked SNPs with -log _10_ mid- *p-valu*es of 7.86 ( Figure 5A) and contained a missense variant in *ARRDC3* codon 182 in exon 4 encoding cysteine (C, tgt) or tyrosine (Y, tat). The homozygous major alleles for the eight best SNPs (and *ARRDC3* Y182) were associated with increased BCHF risk (OR 8.4, CI _95_, 3.3 to 21, Table 2). Conversely, one or two copies of the minor alleles for these eight SNPs (and *ARRDC3* C182) were associated with reduced BCHF risk (OR = 0.12, File S12, *Extended data*). Heterozygosity (i.e., having one risk allele) was also associated with reduced BCHF risk (0.26). The second best associated region spanned a 2.3 kb region in *NFIA* intron 4, and there were no SNPs present on the BovineHD BeadChip array predicted to affect the *NFIA* coding sequence. The 2.3 kb region contained two equivalent linked SNPs with -log _10_ mid- *p-valu*es of 6.60 ( Figure 6). Homozygosity for the major allele of either of these two SNPs was associated with increased BCHF risk (OR 7.4, CI _95_, 2.9 to 19, Table 2). Conversely, one or two copies of the minor alleles for these two SNPs were associated with reduced BCHF risk (OR = 0.14, File S12 *Extended data*). Heterozygosity was also associated with reduced BCHF risk (0.40). Thus, two copies of the major *ARRDC3* and *NFIA* haplotype alleles were associated with BCHF risk.

**Table 2.** Homozygous SNPs associated with the highest risk of BCHF in feedlot cattle.

Genome position ^a^					MAF		McNemar statistics									
Chr	UMD3.1 (bp)	ARS-UCD1.2 (bp)	SNP ID (rs#)	A _1_/A _2_ ^b^	case	control	*b*	*c*	OR	CI _95_	χ ^2^ ^c^	-log( *p*) ^d^	-log( *q*) ^e^	Effect size ^f^	Proportion of informative pairs ^g^	PRS ^h^
7	93229001	90830012	BovineHD0700027233 (rs110586518)	**A**/G	0.18	0.40	42	5	8.4	3.3-21	27.6	7.86	3.09	0.394	0.461	0.18
7	93231670	90832681	BovineHD0700027234 (rs135607340)	**A**/G	0.18	0.40	42	5	8.4	3.3-21	27.6	7.86	3.09	0.394	0.461	0.18
7	93243245	90844254	BovineHD0700027237 (rs109439459)	**A**/G	0.18	0.40	42	5	8.4	3.3-21	27.6	7.86	3.09	0.394	0.461	0.18
7	93243714	90844723	BovineHD0700027238 (rs136675904)	**C**/T	0.18	0.40	42	5	8.4	3.3-21	27.6	7.86	3.09	0.394	0.461	0.18
7	93244933	90845941	BovineHD0700027239 (rs109901274) ^i^	**T**/C	0.21	0.42	42	6	7.0	3.0-16	25.5	7.24	2.71	0.375	0.471	0.18
7	93247780	90848788	BovineHD0700027240 (rs81180002)	**G**/T	0.18	0.39	42	5	8.4	3.3-21	27.6	7.86	3.09	0.394	0.461	0.18
7	93251138	90851664	BovineHD0700027241 (rs3423252075)	**T**/C	0.18	0.40	42	5	8.4	3.3-21	27.6	7.86	3.09	0.394	0.461	0.18
7	93263102	90863630	BovineHD0700027244 (rs3423255708)	**C**/T	0.18	0.40	42	5	8.4	3.3-21	27.6	7.86	3.09	0.394	0.461	0.18
7	93281534	90882079	BovineHD0700027248 (rs3423252071)	**T**/C	0.18	0.40	42	5	8.4	3.3-21	27.6	7.86	3.09	0.394	0.461	0.18
3	85123495	84578325	BovineHD0300024307 (rs136210820)	**G**/A	0.35	0.43	37	5	7.4	2.9-19	22.9	6.60	2.34	0.381	0.412	0.16
3	85125825	84580655	BovineHD0300024308 (rs133192205)	**T**/C	0.37	0.41	37	5	7.4	2.9-19	22.9	6.60	2.34	0.381	0.412	0.16
3	85254825	84707876	BovineHD0300024366 (rs42583839) ^j^	**T** /C	0.25	0.52	44	11	4.0	2.1-7.7	18.6	5.18	1.32	0.300	0.545	0.16

^a^ The SNPs were sorted by chromosome and genomic position. Markers within each genomic region are considered to be statistically equivalent within the margin of error.

^b^ A _1_ was defined as the most frequent allele in the group of 204 cases and controls and is consistent with the ARS-UCD1.2 top strand (antisense strand in *ARRDC3* and *NFIA* genes). The bolded red allele is associated with risk.

^c^ McNemar's chi-squared with continuity correction (see Methods section).

^d^ Log10 of the mid- *p*-value.

^e^ Log10 of the *q*-value.

^f^ The effect size (Cohen's g) for the McNemar test ranges from 0 to 0.5 where 0.5 is the maximum effect. It is the larger value of: the larger of: ( *b*/( *b*+ *c*) or *c*/( *b*+ *c*)) - 0.5.

^g^ The proportion of informative pairs is ( *b*+ *c*)/ *n*.

^h^ The polygenic risk factor value is the (effect size) x (proportion of informative pairs) and represents a value for summing an animal's polygenic risk score.

^i^ SNP BovineHD0700027239 corresponds to *ARRDC3* C182Y (codon tRt) in exon 4 where tGt encodes C182 and tAt encodes Y182.

^j^ SNP BovineHD0300024366 was included for comparison, since it was used in early analyses for obtaining frequency data of U.S. cattle breeds in Table 4.

### Conservation of *ARRDC3* cysteine codon at position 182

**Figure 7.**
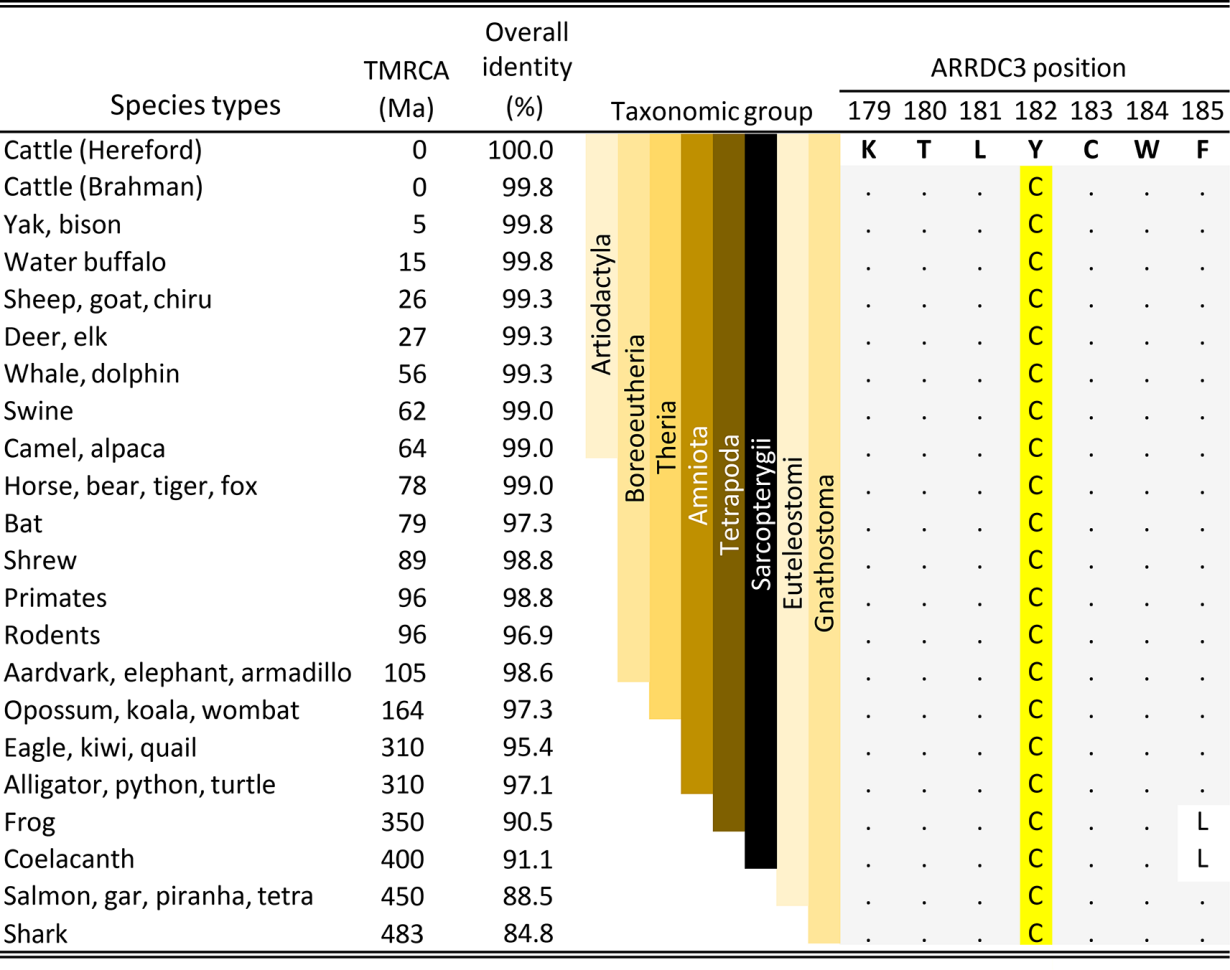
Comparison of ARRDC3 residues near the C182Y position in the Gnathostomes. TMRCA is the estimated time to the most recent common ancestor in millions of years (Hedges *et al.*, 2015). The full length ARRDC3 protein is 414 residues in cattle and most of the Amniota species. The letters are IUPAC/IUBMB codes for amino acids. The dots are amino acid residues identical to those in cattle. A detailed alignment of this region is available in Table S2.

The association of a missense variant in *ARRDC3* with BCHF raised the possibility that the amino acid substitution could affect protein function and thereby increase the risk of disease in feedlot cattle. A comparison with *ARRDC3* codons at position 182 in the jawed-vertebrate species (Gnathostomes) showed the cysteine residue was invariant throughout the 41 representative species examined ( Figure 7). Examples of Gnathostome species with alternative residues at the equivalent position were not observed in any genus. The conservation of the C182 residue throughout the Gnathostomes is consistent with the hypothesis that the C182 residue is critical for normal ARRDC3 protein function and that homozygosity of the *B. taurus*-specific Y182 variant may have a deleterious effect.

### Predicting BCHF risk with *ARRDC3* and *NFIA* SNP genotypes

**Figure 8.**
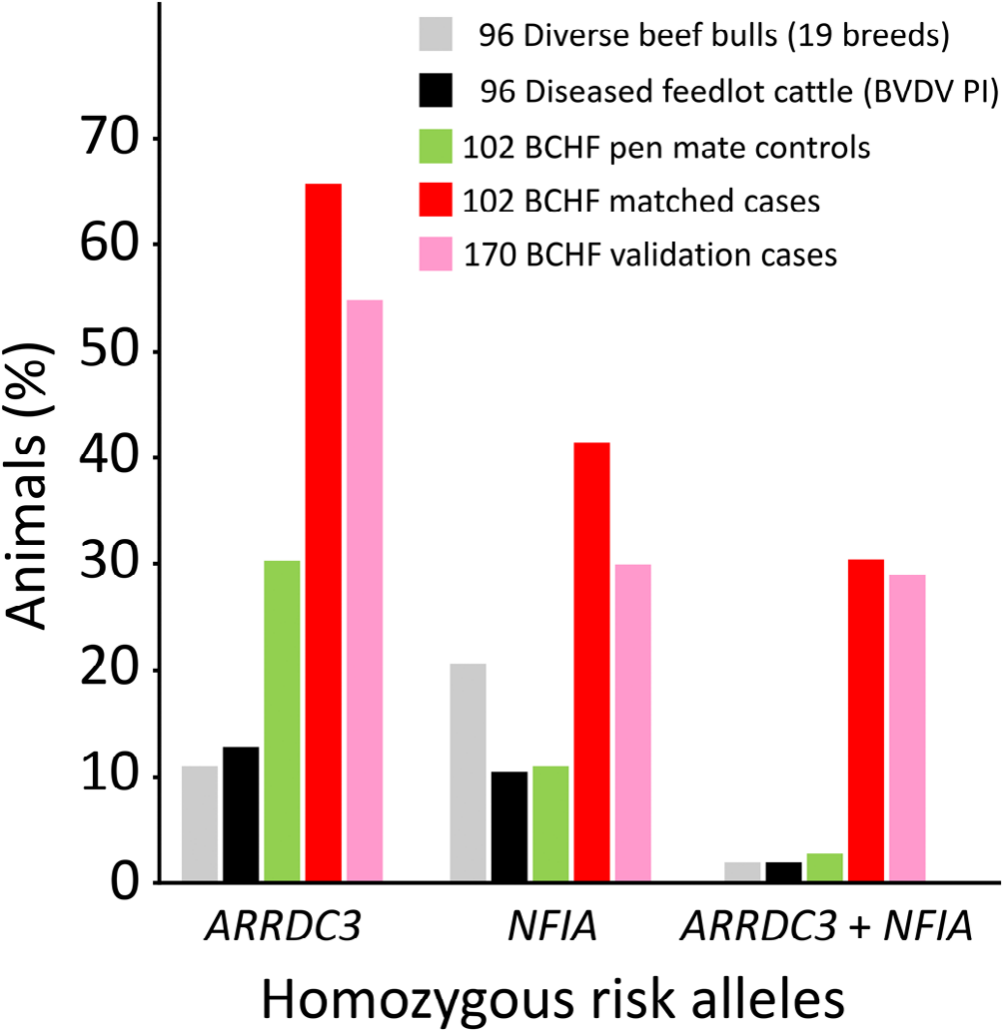
Distribution of homozygous *ARRDC3* and *NFIA* risk alleles in cattle cohorts. Two-SNPs were used for identifying feedlot cattle with homozygous risk alleles (BovineHD0700027239 [ *ARRDC3*] and BovineHD0300024308 [ *NFIA*]). Bovine cohorts: USMARC beef cattle diversity panel v2.9 (19 breeds (Heaton *et al.*, 2016), Kansas feedlot calves persistently infected with bovine viral diarrhea virus (BVDV PI (Workman *et al.*, 2016), pen matched BCHF controls (this study), BCHF cases (this study), independent BCHF validation cases (this study). The BCHF validation cases were collected by pen riders experienced in BCHF diagnosis, however, they were not confirmed at necropsy by veterinarians and researchers. All 204 of the BCHF case-control cattle tested negative for BVDV, and thus were not persistently infected with BVDV.

A custom two-SNP test was designed on a commercially available platform to genotype cattle for the major *ARRDC3* and *NFIA* risk alleles. The platform uses “padlock” oligonucleotide probes, combined with fluorescently labeled probes and isothermal rolling circle amplification technology, for the qualitative detection of alleles. Using reference DNA from animals with known genotypes, the call rate and accuracy typically exceeded 97% and 99%, respectively. This included repeated testing of approximately 10% of the samples due to ambiguous allele calls on the first pass. Using custom genotypes to resolve errors in the original BovineHD BeadChip array, the BCHF OR for calves with homozygous risk alleles at both genes increased to 28-fold more ( *b/c* = 28/1, *p*-value = 1.1 × 10 ^-7^, CI _95_ = 4-206). Testing an independent set of 170 BCHF cases from the same feedlots showed approximately 29% were homozygous for both *ARRDC3* and *NFIA* risk alleles, compared to 29% of the BCHF 102 cases used in the GWAS ( Figure 8). Conversely, an independent set of feedlot cattle persistently infected with BVDV had about 2% homozygosity for both *ARRDC3* and *NFIA* risk alleles, which was similar to the 3% observed with the 102 unaffected animals used in the GWAS. Assuming a 5% BCHF prevalence in the most severely affected pens of feedlot cattle, the adjusted negative and positive predictive values were 0.99 and 0.10, respectively, with a sensitivity and specificity of 0.91 and 0.58. Thus, testing with two SNPs has significant power to identify which animals have low risk for developing BCHF in feedlots, but has little power to predict which animals will develop disease. *ARRDC3* and *NFIA* genotype configurations and their corresponding expected disease and allele transmission risks, together with their allele frequency estimates by breed, are presented in Tables 3 and 4, respectively. Table 5 provides unambiguous genomic identity and sequence context for *ARRDC3* and *NFIA* SNPs associated with BCHF for future genetic testing designs. Although imperfect, these genetic associations and DNA tests provide a starting point for reducing BCHF risk in the most affected herds.

**Table 3.** *ARRDC3* and *NFIA* genotype configurations, expected disease risk, and relative breeding rank.

Possible risk allele configurations ( *ARRDC3*, *NFIA*) ^a^	Total combined risk alleles	Disease risk (1 to 28 scale) ^b^	Probability of risk allele transmission (%)	Breeding rank ^c^
0,0	0	1x	0	1
1,0	1	1x	25	2
0,1	1	1x	25	2
1,1	2	1x	50	3
2,0	2	8x	50	3
0,2	2	8x	50	3
2,1	3	8x	75	4
1,2	3	8x	75	4
2,2	4	28x	100	5

^a^ Diploid combinations of risk alleles for each gene with markers BovineHD0700027239 ( *ARRDC3*) and BovineHD0300024308 ( *NFIA*).

^b^ The risk of developing BCHF in a feedlot with animals and environments like those described here.

^c^ The relative BCHF breeding rank of an animal based on having risk genotypes for *ARRDC3* and *NFIA* from best (1) to worst (5).

**Table 4.** *ARRDC3* and *NFIA* risk allele frequencies in 46 breed groups of US cattle.

		Risk allele frequency ^b^		Animals in each group							
				Disease risk ^c^			Breeding rank ^d^				
Breed group ^a^	Animals typed	*ARRDC3* (BCHF5)	*NFIA* (BCHF2)	1×	8×	28×	1	2	3	4	5
Angus	30	0.72	0.65	9	16	5	0	3	7	15	5
Ankole-Watusi	20	0.00	0.15	19	1	0	15	4	1	0	0
Ayrshire	24	0.02	0.50	17	7	0	7	9	8	0	0
Beefmaster	29	0.33	0.67	13	16	0	1	6	14	8	0
Belgian Blue	24	0.19	0.85	7	17	0	0	7	8	9	0
Blonde d'Aquitaine	24	0.06	0.42	19	5	0	9	8	6	1	0
Brahman	29	0.00	0.05	29	0	0	26	3	0	0	0
Brahmousin	24	0.00	0.29	22	2	0	12	10	2	0	0
Brangus	29	0.45	0.55	17	10	2	2	8	9	8	2
Braunvieh	28	0.00	0.50	21	7	0	7	14	7	0	0
Brown Swiss	24	0.00	0.50	18	6	0	6	12	6	0	0
Charolais	30	0.05	0.48	24	6	0	7	15	7	1	0
Chianina	28	0.18	0.54	17	10	1	5	10	10	2	1
Corriente	27	0.00	0.59	18	9	0	4	14	9	0	0
Devon	23	0.39	0.26	16	7	0	5	9	6	3	0
Dexter	24	0.00	0.31	23	1	0	10	13	1	0	0
Gelbvieh	29	0.03	0.48	20	9	0	9	11	8	1	0
Guernsey	24	0.00	0.56	19	5	0	2	17	5	0	0
Hereford	30	0.58	0.50	13	17	0	2	2	15	11	0
Highland	24	0.00	0.27	23	1	0	12	11	1	0	0
Holstein	23	0.09	0.63	16	7	0	1	12	9	1	0
Indu-Brazil	24	0.00	0.00	24	0	0	24	0	0	0	0
Jersey	38	0.00	0.58	24	14	0	8	16	14	0	0
Limousin	30	0.12	0.40	24	5	1	7	17	5	0	1
Maine-Anjou	29	0.24	0.76	13	15	1	0	7	16	5	1
Marchigiana	23	0.00	0.61	11	12	0	7	4	12	0	0
Mini Hereford	24	0.58	0.73	7	12	5	0	5	4	10	5
Mini Zebu	24	0.00	0.04	24	0	0	22	2	0	0	0
Montbeliard	24	0.08	0.42	21	3	0	7	11	5	1	0
Murray Gray	24	0.71	0.42	11	11	2	1	5	7	9	2
Nelore	24	0.00	0.31	23	1	0	10	13	1	0	0
Piedmontese	24	0.00	0.48	20	4	0	5	15	4	0	0
Pinzgauer	24	0.00	0.44	19	5	0	8	11	5	0	0
Red Angus	30	0.73	0.42	12	16	2	0	5	13	10	2
Red Poll	24	0.48	0.48	16	5	3	5	2	10	4	3
Romagnola	24	0.00	0.48	21	3	0	4	17	3	0	0
Salers	29	0.09	0.36	23	6	0	12	8	9	0	0
Santa Gertrudis	28	0.18	0.43	24	3	1	4	18	3	2	1
Senepol	24	0.02	0.65	15	9	0	2	12	10	0	0
Shorthorn	29	0.24	0.57	17	12	0	2	12	10	5	0
Simmental	30	0.13	0.42	23	6	1	9	13	5	2	1
Tarentaise	28	0.09	0.20	27	1	0	14	12	2	0	0
T. Longhorn, MARC	28	0.02	0.41	23	5	0	10	13	4	1	0
T. Longhorn, CTLR	23	0.04	0.61	14	9	0	3	10	10	0	0
Tuli	23	0.00	0.17	23	0	0	15	8	0	0	0
Wagyu	24	0.06	0.73	10	14	0	2	8	12	2	0

^a^ Registered cattle chosen for minimal pedigree relationships (Heaton *et al.*, 2016). Breed groups were genotyped from whole genome sequence (WGS) mapped to the bovine reference genome assembly (ARS-UCD1.2).

^b^ The risk allele definitions are listed in Table 2. The sites genotyped from WGS data were chr7:90845941 (BCHF5) and chr3: 84707876 (BCHF2).

^c^ As described in Table 3.

^d^ As described in Table 3.

**Table 5.** Genome sequence context for *ARRDC3* and *NFIA* SNPs associated with BCHF.

Genome position				SNP identifier					Flanking features in 19 beef breeds ^d^			
Chr ^a^	UMD3.1 (bp)	ARS-UCD1.2 (bp)	Gene	Bead Array	rs number	Alias	A _1_/A _2_^b^	200 bp flanking genome sequence ^c^	Type	ARS-UCD1.2 (bp)	Diff. (bp)	MAF
7	93229001	90830012	*ARRDC3*	BovineHD0700027233	rs110586518	BCHF60	**A**/G	5′-TACTCCAGAGTATCAGGGACCCATGAGGAATGATCAAGAAATGAAAATGGGGGAAAATGGGATTTTAAAATGGGTGGAAAGAAGTAAG **M**ATTGGGTGAGA **[ A/G]** GATGAAAACCTAGACGAGGAGGGTAGCAACGGGAGTGGAAGAACAGGGACAGGGACCCTGGAGATCTTACGAGAAGACCGAGGAAACTTCATACCTTACT-3′	SNP SNP	90830000 90830012	-12 0	0.281 0.281
7	93231670	90832681	*ARRDC3*	BovineHD0700027234	rs135607340	BCHF61	**A**/G	5′-CTTAGAGAACGAAACGAGTTAATAGATTTTCCTCAAATTGGAGCAATTTTCTTCTTCTTTATTGAATCAGGAAAGCTAACT **R**TCAAAGAGGCATAGAGTG **[ A/G]** CGTAGATAATGCCCTAAGGTAACAAAACTCTATTATGTTGTGTTACATTAGCACCGAGCGAAGGGTATTTTA **R**AAGGAAATTTCACTGTGTAATAAGGGG-3′	SNP SNP SNP	90832662 90832681 90832754	-19 0 +73	0.083 0.281 0.083
7	93243245	90844254	*ARRDC3*	BovineHD0700027237	rs109439459	BCHF33	**A**/G	5′-TAAGAGACCAGTCCAATGCTGGGTCAGACTTTGTATTAAGTATTGACAATCTAACTTTTAATCTGTTGTTCAAAATTATTTATTTCTCTCTTACCATGAT **[ A/G]** ATCAAAACCTAACTGTATTTTCAGAAACTCTGCAATATTGCTTAGTAAGAGTCAGCCCATTTTGTACTTGAGGGTGTGATGCTTACCTCTGAATAAAGGG-3′	SNP	90844254	0	0.281
7	93243714	90844723	*ARRDC3*	BovineHD0700027238	rs136675904	BCHF34	**C**/T	5′-GGAAATATACATTTTAAATCTAACATTAGGGGCACACCCCATTCTTCAATACAGACAAATTTTATCTAATCATAAAATATCAGAAGCAAAATTATTTTCT **[ C/T]** GCAAATACCAGATTAAGCTTAGATACGCATTATCAAATTATTACCTTCAGGTCTTTCAGGTAGGGATAAACCAAGCCAGTTCATGTTCATGCTACACTGA-3′	SNP	90844723	0	0.281
7	93244933	90845941	*ARRDC3*	BovineHD0700027239 ^e^	rs109901274	BCHF5	**T**/C	5′-TCAGTCTTGGTGGGAGTCCACCTCATACGTACCTGGCGTATAGCCCTTCCTTTCGATCTTGGCACTTAAGGATATGGGGCCTGAGGTACAGAACCAGCAA **[ T/C]** AGAGAGTCTTTTCTTTTGTGCCTGCTTGGGGTGACTGTAAGATATAAATTTAGAG **R**AAAAAATCAAGGTATGAATTGAGACAGGAATAAAATTTTTAAAA-3′	SNP SNP	90845941 90845997	0 +56	0.224 0.177
7	93247780	90848788	*ARRDC3*	BovineHD0700027240	rs135153741	BCHF35	**G**/T	5′-ATGACAGACTTGATATGTTCTCTTTTTTCAGCTAATGCTGATGGTGAACTATTAAGTGCTTACTATTTGACTAGCACCTTCCAATTCATTCACTTTAAAT **[ G/T]** AAATACTTTTAAACACCATACAAATCCTGGGACGGAATATTGAGGTTTGCTTTCTATAATTTTGCCACTCCAGATTAAAAAATAGCTT **Y**TTTTTTTTTAG-3′	SNP SNP	90848788 90848877	0 +89	0.412 0.068
7	93251138	90851664	*ARRDC3*	BovineHD0700027241	rs110066813	BCHF36	**T**/C	5′-GTTTGTCCCCGTAAGTCGTATTATTATTCTACTTTTACCCCACTGCACACCACAACTCCTTAAAAACCTGTTTCTTCTAACCCATCTTAAAAAAAGCTAC **[ T/C]** AGTCAAGGAAGGATCAAATACAAACTCCTGGGCGTCCTGTTCAGCCAATACCAGGACTGTCATTAGGCAGTTCCATTTCTTATGACTGTCAGTGCTTGGA-3′	SNP	90851664	0	0.260
7	93263102	90863630	*ARRDC3*	BovineHD0700027244	rs109860522	BCHF62	**C**/T	5′-CAGTGTTTAATCTGTCAGTGTAGGACTCATTAATTGTTGTTGTTGAATTATTCTTTTTTATTTCAGTAGGGCTTCCTTTCCCAAAATAATGTTTAGTTAT **[ C/T]** AATAAACAACTCTTTACGTGATTGAAGACATTGTTTTCCACAAATTTATACATTTTGCCCCCAGATATGTTTAAAGTATATATATGCAC **Y**ATATTTGCTA-3′	SNP SNP	90863630 90863720	0 +90	0.260 0.021
7	93281534	90882079	*ARRDC3*	BovineHD0700027248	rs110253449	BCHF63	**T**/C	5′-TTCTTTTTGCCTGG **K**GCTGCACAGATCTTGAAGTCACTGCCAAACTAACTCTGCAGTGGCAACTTCAGAACACAAAATAAGCACTAGACCTAGAAATGCT **[ T/C]** GACACCCAAACTTAACGTATAGTCAAATAAATGGTTTAGTCTAGCACCTACCAGCCTTTAAGAAGAGCTTGTCAGTATACTCTTTCCTGTTGAAAGGAAA-3′	SNP SNP	90881993 90882079	-86 0	0.026 0.276
3	85123495	84578325	*NFIA*	BovineHD0300024307	rs136210820	BCHF31	**G**/A	5′-TTTTCCCTGGA **W**GAAATGAACTGCTGACATTCCGTGAACTCTATCTTTGCCTCACTTCTCACCCTGACCGTCAAACAAAGAAATCTGTCTTGAGTTCCAC **[ G/A]** ATGTACCCCATGTCACTATGACTAATCCAAAGCATTTTTATTTACAGTGTTCAGAAT **R**AATTTCTATGACAATCTCATGCCATTCTCACGTGCCTTTTGA-3′	SNP SNP SNP	84578236 84578325 84578383	-89 0 +58	0.042 0.448 0.052
3	85125825	84580655	*NFIA*	BovineHD0300024308	rs133192205	BCHF32	**T**/C	5′-TTACTAACCATGGACCAAGGACACATTTCTCAGCCTCTTTGTTTCTCAGCAGCATTAAGGCAATTATAAAAGCTCCCCTATCTAAAAGGTAAGATTTACA **[ T/C]** GAAGAACTAATAAAATAATGTATAAGAACCAACTTAAATATCATAGAAATGAACAACACTAGTCTAGCAGATCTGGCTTTCATCCTGGCTCTGCCACTTA-3′	SNP	84580655	0	0.422
3	85254825	84707876	*NFIA*	BovineHD0300024366 ^f^	rs42583839	BCHF2	**T**/C	5′- **tatctgcatgcaatgcaggagacctgggttcaatccctgggtcaggaagatcccctggaggagggcatggcaa** TACTTCTGCTCTTGCTTAGAATAAAAT **[ T/C]** CAGTTCCCATGACAACCTGGTGGCCCTGAGGAG **M**CCGAAC **R**ACTCCTGCTGCAGGGACCTGACCCTGTTCCGCTGCCTCCCTGTACATTCTGATCTGCGG-3′	SNP SNP SNP	84707876 84707910 84707917	0 +34 +41	0.464 0.021 0.073

^a^ Chromosome.

^b^ A1 was defined as the most frequent allele in the group of 204 BCHF cases and controls and the orientation is consistent with the ARS-UCD1.2 top strand (antisense strand in *ARRDC3* and *NFIA* genes). The bolded red allele is associated with risk.

^c^ Sequence orientiation is consistent with the ARS-UCD1.2 top strand. Each sequence aligned uniquely to the reference genome except BovineHD0300024366 (rs42583839) which contained 75 bp of repetative sequence denoted in lowercase (blue).

^d^ Derived from WGS data with USMARC beef cattle diversity panel version 2.9 (MBCDPV2.9, Heaton *et al.*, 2016), and PLINK software (v1.9, Chang *et al.*, 2015) with filters for MAF greater than or equal to 0.015, and missing genotypes less than or equal to 0.05.

^e^ SNP BovineHD0700027239 corresponds to *ARRDC3* C182Y (codon tRt) in exon 4 where tGt encodes C182 and tAt encodes Y182.

^f^ SNP BovineHD0300024366 was included for comparison, since it was used in early analyses for obtaining frequency data of U.S. cattle breeds in Table 4.

## Discussion

The present report describes a GWAS with clinical cases of BCHF and their pen matched controls from feedlots in the Western Great Plains. This is a region of the U.S. where outbreaks can be severe and occur annually. The 102 clinical cases enrolled in this study were a subset screened by pen riders from more than 140,000 feedlot cattle over the span of 16 months and were derived from 30 ranch sources. The most significant genome-wide associations were limited to two regions containing *ARRDC3* and *NFIA* genes, respectively. In each region, multiple linked SNPs were associated with BCHF in feedlot cattle. It is unknown whether the associated SNPs are merely linked to variants responsible for the elevated BCHF risk, or whether they are involved directly in pathogenesis. Whole genomic sequence (WGS) analyses are underway for the 102 case-control pairs of animals and may unlock further insights in the future. Despite the lack of resequencing data in the present study, animals having two copies of risk alleles in either gene, or both genes, were eight- and 28-fold more likely to develop BCHF, respectively. Thus, specific DNA sequence variants within *ARRDC3* and *NFIA* are markers for BCHF risk in feedlot cattle in the Western Great Plains, either through linkage to causal variants or direct effects. Neither gene was previously reported to be a direct cause of heart failure in animals. However, both genes play roles in the complex biology of cardiovascular health.

*ARRDC3* is a member of the alpha arrestins family involved in regulating mammalian metabolism. The normal biological functions of *ARRDC3* in cattle have not been studied, however variants have been associated with bovine growth and conformation traits ( Saatchi *et al.*, 2014; Bolormaa *et al.*, 2014; Seabury *et al.*, 2017; Abo-Ismail *et al.*, 2017; Jiang *et al.*, 2019; Smith *et al.*, 2019). Studies on humans and mice have shown that *ARRDC3* has numerous functions. It regulates G protein-coupled receptor trafficking and signaling in cancer, suppresses tumors, is differentially regulated by ubiquitin, and functions as a multifaceted adaptor protein to control protein trafficking and cellular signaling ( Wedegaertner *et al.*, 2023). The ARRDC3 protine interacts with at least five membrane receptors or transporters, including: β2- and β3-adrenergic receptors (β2AR and β3AR), protease activated receptor-1 (PAR1), insulin receptor (IR), and integrin beta 4 (Itgβ4) ( Wedegaertner *et al.*, 2023). *ARRDC3* has also been linked to regulation of adrenergic signaling through interaction and regulation of ubiquitination and trafficking of the β2AR ( Han *et al.*, 2013; Tian *et al.*, 2016). The β2AR is present on the cell surface and its stimulation causes smooth muscle relaxation, which may result in peripheral vasodilation bronchodilation with subsequent hypotension and reflex tachycardia.

*ARRDC3* can also signal activation of uncoupling protein-1 and the production of heat when thermogenesis is needed during cold stress. Over-activation of human β2AR promotes cardiac hypertrophy and represents an independent risk factor for cardiovascular morbidity and mortality ( Osadchii, 2007). *ARRDC3* also modulates insulin action and glucose metabolism in the liver and binds directly to the insulin receptor in a mouse model for human obesity ( Batista *et al.*, 2020). In humans, increased *ARRDC3* expression was associated with an increase in body mass index, while *ARRDC3* deficient mice were resistant to age-related obesity and have increased insulin sensitivity ( Patwari *et al.*, 2011). However, 75% of the *ARRDC3* deficient mice did not survive to weaning. Although it is tempting to link human *ARRDC3* obesity mechanisms with the process of fattening cattle, more than half the BCHF cases in our study died in the first, less intensive phase of the 240-day feeding program. This is inconsistent with the hypothesis that fattening is the underlying cause of disease in these cattle ( Heaton *et al.,* 2019). Regardless, biological mechanisms by which genetic variation in the *ARRDC3* genomic region affects BCHF are presently unknown.

**Figure 9.**
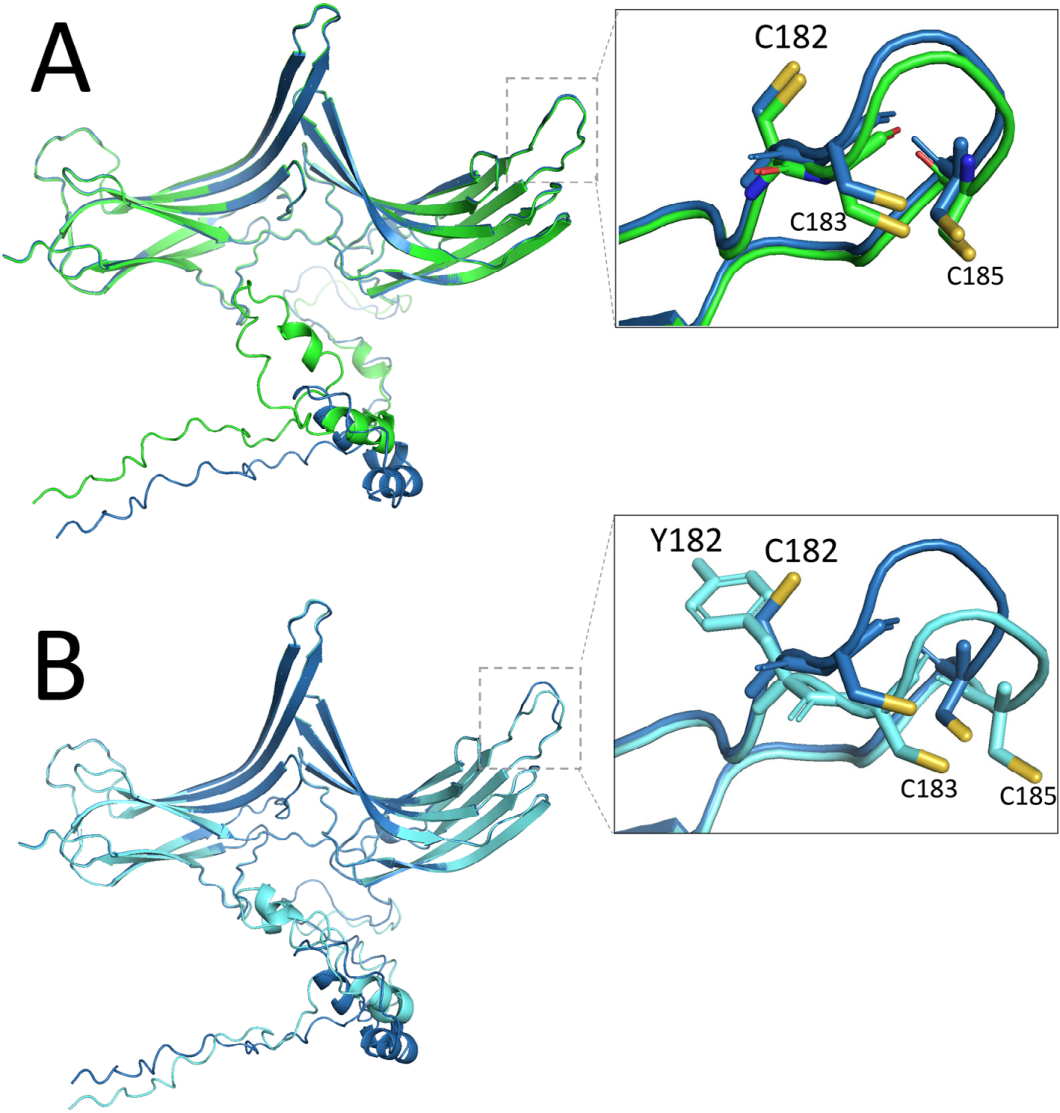
Predicted protein folding differences between bovine ARRDC3 C182Y variants. Alignment of ribbon representations of AlphaFold2 predictions of bovine ARRDC3. Panel A, human and bovine ARRDC3 with inset showing the side chains of the C182, C183 and C185 residues. Panel B, bovine ARRDC3 with C182 or Y182 residues with inset showing the sidechains of the variant C182 and Y182 residues, together with invariant C183 and C185 residues.

We hypothesize that the Y182 missense variant negatively alters the normal ARRDC3 protein functionality but does not eliminate it. The human and bovine ARRDC3 protein sequences are 99% identical and their aligned tertiary structures from *de novo* structure predictions shows they are nearly superimposable ( Figure 9A). The ARRDC3 C182 residue is part of a three-cysteine loop at the end of two antiparallel beta sheets in the Ig-like beta-sandwich folds at the beginning of the Arrestin-C domain ( Figure 9A inset). We speculate that the bovine Y182 variant alters the normal ARRDC3 function in a way that is deleterious to the individual. This hypothesis is based on the observation that all jawed vertebrates tested have cysteine exclusively at this position. Since the Gnathostomes represent 99% of all living vertebrate species (Brazeau and Friedman, 2015), it is remarkable that only cattle are reported to encode a residue other than cysteine at this position. We also speculate that there may be residual function in bovine *ARRDC3* Y182 based on two observations: 1) *ARRDC3* knockout mice are lowly viable and have a range of perinatal developmental abnormalities including heart and lung defects ( La Marca *et al.*, 2023), and 2) homozygous healthy cattle with the non-conserved Y182 genotypes are abundant (this report). However, further research is needed to reject the hypotheses presented here.

*NFIA* is a transcription factor that controls various cellular differentiation programs, including myogenesis and adipogenesis ( Hiraike *et al.*, 2020). The only biological function of *NFIA* in cattle was vitamin A effects on *NFIA* expression levels in brown fat depots ( Chen *et al.*, 2018), and no prior bovine GWAS has linked *NFIA* to any traits *.* In humans, *NFIA* was associated with heart failure, reduced ejection fraction, and conduction of the electrical signal throughout the ventricular myocardium ( Joseph *et al*., 2022; Evans *et al.*, 2016). *NFIA* also controls the onset of gliogenesis in the developing spinal cord in mice ( Deneen *et al.*, 2006). *NFIA* is best known for activating adipogenesis, as well as actively suppressing myogenesis by binding to brown-fat-specific enhancers before differentiation, and later facilitates the binding of the master transcription factor of adipogenesis: peroxisome proliferator-activated receptor γ ( *PPARγ*) ( Hiraike *et al.*, 2017). A reduction in *NFIA* activity shifts the balance towards the myogenesis and white adipocyte gene program, while an increase in *NFIA* activity shifts the balance towards the brown/beige adipocytes program ( Hiraike *et al.*, 2020). Thus, it is tempting to speculate that *NFIA* variants in beef cattle have been selected for reduced activity leading to increased muscle and white fat; however, there is no evidence for this.

Still, an intriguing but unproven connection may exist between *ARRDC3* regulation of β-adrenergic signaling and *NFIA* functions relating to cold stress and brown fat thermogenesis. Anecdotally, the incidence of feedlot BCHF increases dramatically each year during the first wave of bitter cold weather, when many animals succumb to BCHF in a short period of time (Personal communication between authors (MPH and BLVL) and Nebraska feedlot owners). In any event, the biological mechanisms explaining how either of these genes may influence BCHF pathogenesis remain to be determined.

A strength of this study was the diagnosis and necropsy of clinical cases at the time of euthanasia. This was accomplished immediately, in the environment and geographic region where the disease is most apparent, and included feedlot pen riders with experience in identifying animals displaying disease signs. The case definition at necropsy was rigorous and included multiple specific and non-specific clinical signs to eliminate other potential confounding afflictions. This is in contrast to case definitions based on qualitative cardiomegaly scores obtained during beef processing of animals passing federal antemortem inspection. Cases described in the present study were deemed by pen riders unlikely to survive transport to a processing plant, and even less likely to pass federal inspection, hence their availability for euthanasia and necropsy.

Another strength was that feedlot personnel were incentivized to monitor more than 140,000 head from representative feedlots to find those animals most likely to have terminal BCHF and euthanize them before they died of congestive heart failure. The financial loss of each case enrolled in our study was offset by a $500 indemnity payment when the animals were identified early, euthanized, and necropsied by the authors. Additional strengths of this study included the use of: diverse ranch sources, matched controls, and custom McNemar allele analysis. Approximately 20% of the collected cases were dismissed due to inadequate pair-matching criteria. Weaknesses of the study included the relatively low number of total pairs (102) that met the qualifications for the study. However, the cost of indemnifying more clinical cases, combined with the time and resources needed for researchers to conduct careful necropsies and sample collections significantly affected the feasibility of going much beyond 100 matched pairs. Ideally, the study would have benefited from 200 matched pairs for discovery, and another 200 matched pairs for validation. Our validation set of 170 clinical cases consisted of unmatched cases that were either animals suspected of having BCHF and shipped early to a salvage beef processor, or those that died in the feedlot.

Genetic testing of young cattle from affected herds can identify animals predisposed to developing BCHF in Western Plains feedlot environments. Using a two-SNP test with markers for *ARRDC3* and *NFIA* risk alleles, BCHF cases are expected to occur in animals with homozygous *ARRDC3* and *NFIA* risk alleles at a rate 28-fold higher than those without homozygous risk alleles in either gene. Although the confidence interval around this OR estimate is wide (four- to 206-fold), the test provides a starting point for applying costly BCHF interventions to a smaller group of animals rather than the whole pen. For example, a subset of high-risk animals could be moved to a lower elevation for fattening, or they could be fattened with less energy-dense feed for a longer interval based on genetic testing. The two-SNP test used here has a remarkably high negative predictive value (0.99) that is powerful for identifying sires without these major risk alleles. Using low-risk sires and either conventional breeding or artificial insemination would have an immediate impact on reducing BCHF in the next generation of calves.

The high frequencies of *ARRDC3* and *NFIA* risk alleles in some affected breeds (> 0.65) raise the question of why the disease prevalence is relatively low even in outbreaks (> 0.07)? This phenomenon may be due to the multifactorial nature of complex disease. The development of BCHF in cattle is presumed to be influenced by exposure to non-genetic factors, such as elevation (hypoxia), extreme weather conditions, feeding schedules, ionophores, feed grade antibiotics, steroid hormone implants, and beta-adrenergic agonist feed additives ( Krafsur *et al.*, 2019; Neary *et al.* 2018). These environmental and management factors may play significant but unknown roles in disease development. In addition, most complex diseases are considered polygenic with variation at many genes having small effects ( Khera *et al.* 2018). DNA sequence variation identified here in *ARRDC3* and *NFIA* genomic regions may predispose cattle to BCHF under certain conditions, despite the absence of any known causal pathogenic mutations.

The high frequencies of risk alleles observed in some breeds also poses a challenge for selective breeding. Although estimates for the positive predictive value of the *ARRDC3* and *NFIA* markers were low (0.10), the negative predictive value was quite high (0.99). Consequently, identifying breeding stock lacking these risk factors could prove useful for reducing a herd's overall risk. The challenge is finding sires without the *ARRDC3* and *NFIA* risk alleles in breeds with a high prevalence of high-risk alleles. For instance, the largest affected breed in the United States, American Angus, has more than 300,000 bulls registered annually in its association. However, less than 5% (15,000) are expected to possess the optimal low-risk genotypes at both the *ARRDC3* and *NFIA* gene regions (M. Heaton, L. Kuehn, and B. Vander Ley 2022, unpublished). To identify the best bulls for decreasing BCHF risk alleles in their herds, cattle producers may need to obtain genotypes from hundreds of candidate sires. Unfortunately, this information is not widely available for most bulls at present. Despite these challenges, there are a number of reasonable selection strategies that could help reduce the risk of BCHF in the long term, depending on the specific goals and circumstances of the cattle producer.

Identification of the most significant genome-wide markers for BCHF was based on the FDR results presented in Figure 4. Only models that compared homozygous risk alleles against all other genotypes yielded significant results. The choice of best markers for genetic testing was based on results presented in Table 2 and Figure S2. On chromosome 7 in the 52 kb associated genomic region centered on the 14kb *ARRDC3* gene sequence, there were eight SNPs linked to the C182Y missense variant (r ^2^ = 0.87 to 0.91). The difference in the OR based on informative McNemar’s pairs between the eight linked SNPs and the C182Y variant was not significant ( *b/c* = 42/5 and 42/6, respectively). Thus, any of the eight linked SNPs could be used as a proxy for the C182Y SNP in exon 4 until a variant(s) directly responsible for the elevated BCHF risk is confirmed in this region. In the 2.32 kb associated genomic region of *NFIA* intron 4 there were two linked SNPs (r ^2^ = 0.94) with high statistical association with BCHF and identical McNemar’s statistics ( *b/c* = 37/5, CI _95_ = 2.9-19, BovineHD0300024307, BovineHD0300024308). Both *NFIA* intron 4 SNPs were in moderate linkage disequilibrium with a third SNP located 127 kb upstream in *NFIA* antisense ( *AS*) 2 region (r ^2^ = 0.49 and 0.45, respectively). The *NFIA AS2* SNP (BovineHD0300024366) had more informative pairs which corresponded to a smaller confidence interval ( *b/c* = 44/11 and CI _95_ = 2.1-7.7, respectively), but the OR was lower (4.0). Thus, the two *NFIA* intron 4 SNPs are nearly as informative, yet have a better OR (7.4). A minimum two-SNP assay encompassing most of the association would include the *ARRDC3* C182Y and a NFIA intron 4 marker ( *e.g.*, BovineHD0700027239 [alias BCHF5] and BovineHD0300024308 [alias BCHF32], respectively).

Future prospects for improved BCHF genetic markers are promising. The 560,000 BovineHD BeadChip array SNPs in the present GWAS represent less than 5% of 12 million variants in the Angus genome ( Low *et al.*, 2020). Thus, WGS analysis of the 102 case-control pairs would refine the bounds of the genomic region most strongly associated with BCHF and identify the complete set of tagging SNPs appropriate for use in genotyping by sequencing. Any novel variants with increased association would potentially represent candidates for functional variants conferring BCHF risk. A WGS analysis may also identify any newly associated genomic regions previously missed due to ascertainment bias in the SNP selection for the BovineHD BeadChip array. For these reasons, we are endeavoring to produce and analyze 15-fold WGS coverage from these 204 animals. Additional markers from new prospective associated regions could also potentially be used in genetic tests to calculate polygenic risk scores for individual animals, and further improve disease risk prediction by stratifying populations into risk groups ( Knowles and Ashley, 2018; Torkamani *et al.*, 2018; Wise *et al.*, 2019; Claussnitzer *et al.*, 2020). Thus, a two-SNP test for *ARRDC3* and *NFIA* risk alleles presented here represents a first genetic tool for identifying an animal’s genetic risk for BCHF and may be helpful in determining whether the *ARRDC3* and *NFIA* markers are also associated with the high-elevation “brisket disease” disorder in the Rocky Mountain region of Colorado and Utah. Regardless of the outcome, additional DNA markers may be needed to increase risk prediction accuracy for BCHF in feedlot cattle.

## Conclusions

DNA sequence variation in *ARRDC3* and *NFIA* was discovered to be associated with BCHF in feedlot cattle at moderate elevations. The role of these variants is unknown and could be either through linkage to causal variants or direct effects on pathogenesis. Animals that were homozygous for risk alleles in both genes were 28-fold more likely to develop heart failure than those without. A custom DNA-based test showed 29% of clinical cases had homozygous risk alleles in both genes, compared to less than 2.5% in 198 similar unaffected feedlot cattle. This type of test may be useful for identifying feedlot animals at the highest risk for BCHF in the Western Great Plains of North America. In herds affected by BCHF, knowledge of which cattle have the highest and lowest genetic risk for disease allows producers to make informed decisions for selective breeding and animal health management.

## Data availability

### Underlying and extended data

Figshare: Additional files for the article entitled, Association of *ARRDC3* and *NFIA* variants with bovine congestive heart failure in feedlot cattle, https://doi.org/10.6084/m9.figshare.c.4331114.v2 ( Heaton, 2022).

This project contains the following data files:

• Table S1. Metadata for 102 BCHF cases and their matched controls
• Table S2. Extended comparison of ARRDC3 residues near the C182Y position in the Gnathostomes. https://figshare.com/articles/dataset/Table_S2_Extended_comparison_of_ARRDC3_residues_near_the_C182Y_position_in_the_Gnathostomes_/22213495
• Table S3. *ARRDC3* and *NFIA* genotypes for 204 case-control animals corresponding to 12 SNPs listed in Table 2. https://figshare.com/articles/dataset/Table_S3_i_ARRDC3_i_and_i_NFIA_i_genotypes_for_204_case-control_animals_corresponding_to_12_SNPs_listed_in_Table_2_/24986211
• Table S4. *ARRDC3* and *NFIA* genotypes for two SNPs from the 566 animals shown in Figure 8. https://figshare.com/articles/dataset/Table_S4_i_ARRDC3_i_and_i_NFIA_i_genotypes_for_two_SNPs_from_the_566_animals_shown_in_Figure_8/24986424
• Table S5. *ARRDC3* and *NFIA* genotypes for two SNPs from the 1203 animals and 46 breeds listed in Table 4. https://figshare.com/articles/dataset/Table_S5_i_ARRDC3_i_and_i_NFIA_i_genotypes_for_two_SNPs_from_the_1203_animals_and_46_breeds_listed_in_Table_4_/24986442
• Figure S1. Calculation of expected theoretical *q*- and mid *p*-values
• Figure S2. Linkage disequilibrium between SNPs associated with BCHF in *ARRDC3* and *NFIA*
• Files S1 and S2. Unfiltered map and ped files (777,962 SNPs) for 102 BCHF matched case-control pairs, filename: BCHF102pairsHD778 (28.9 and 605.4 Mb, respectively.)
• Files S3 and S4. Filtered map and ped files (563,042 SNPs) for 102 BCHF matched case-control pairs, Filename: BCHF102pairsHD770Filtered (20.9 and 438.2 Mb, respectively.)
• Files S5 and S6. Unfiltered map and ped files (777,962 SNPs) for 102 BCHF pairs and 96 MBCDPv2.9 bulls, filename: BCHF102_MBCDP29_HD778 (27.6 and 890.3 Mb, respectively.)
• Files S7 and S8. Filtered map and ped files (604,746 SNPs) for 102 BCHF pairs and 96 MBCDPv2.9 bulls, filename: BCHF102_MBCDP29_HD778Filtered (21.5 and 692.1 Mb, respectively.)
• Files S9 and S10. Filtered map and ped files (563,087 SNPs) for 102 BCHF matched case-control pairs, filename: BCHF102pairsHD778FFFSortFiltered (20.9 and 438.2 Mb, respectively.)
• File S11. Unformatted McNemar output file (563,087 SNPs) for 102 BCHF matched case-control pairs, filename: BCHF102pairsHD778FFFSortFiltered.McNemarsResults.csv (468.74)
• File S12. McNemar’s test statistics for 561,683 filtered SNPs, filename: BCHF102PairsHD778FFFSortFiltered.McNemarsResults17.xlsx (450.8 Mb)
• File S13. Genome-wide SNPs passing the 5% FDR. Filename: BCHF102PairsHD778FFFSortFilteredExtractTwoHF_q_LT_0_05_MPH3.xlxs (90k)

Data are available under the terms of the Creative Commons Attribution 4.0 International license (CC-BY 4.0).

### Analysis code

MatLab analysis code available at GitHub: https://github.com/greg-harhay/McNemarsSNPAnalysis

Executable MatLab code available at Code Ocean: https://doi.org/10.24433/CO.0870729.v1

Data are available under the terms of the UnLicense.
