# Supplementary material for: Association of ARRDC3 and NFIA variants with bovine congestive heart failure in feedlot cattle
Source: F1000Res. 2024 Mar 4;11:385. Originally published 2022 Apr 1. [Version 2] doi: 10.12688/f1000research.109488.2 (PMC11046187; doi:10.12688/f1000research.109488.2)
Supplement: Peer review history

# Reviewer report: Reviewer response for version 2

**DOI:** 10.5256/f1000research.162702.r252387

**Published:** 2024-07-24

**Authors:** Cord Drögemüller

**Affiliations:** 1 Institute of Genetics, Vetsuisse Faculty, University of Bern, Bern, Switzerland

What is known about the possible inheritance of BCHF? Have segregation analyses been performed? What is the heritability?

 The nomenclature of variants, especially the representation of ARRDC3 alleles, should necessarily follow the current recommendations, see Richards S, et.al.,2015 [Ref 1]

 The intronic variant in the NFIA gene remains functionally absolutely unclear, therefore talk about a linked marker rather than a possible causal variant.

 The wording "homozygous major alleles" on page 10 is unfortunate. Would it not be better to say homozygous for the major allele. An allele itself cannot be homozygous, but the genotype is either hetero- or homozygous for two or one allele.

 In Figure 7 the accession numbers of the listed protein sequences are missing.

 Table 4 would be easier to understand if actual genotype distributions were listed. Also missing is a clear table on the genotype distribution of the two highlighted markers for the animals from the discovery and confirmation cohorts to clearly assess the results.

 Table 3 is then a further transformation towards possible practical application, but this original publication should include the true genotypes clearly and legibly.

 I would be happy to if authors could address these minor revisions.

Is the work clearly and accurately presented and does it cite the current literature?

Yes

If applicable, is the statistical analysis and its interpretation appropriate?

I cannot comment. A qualified statistician is required.

Are all the source data underlying the results available to ensure full reproducibility?

Yes

Is the study design appropriate and is the work technically sound?

Yes

Are the conclusions drawn adequately supported by the results?

Partly

Are sufficient details of methods and analysis provided to allow replication by others?

Yes

Reviewer Expertise:

Animal genetics

I confirm that I have read this submission and believe that I have an appropriate level of expertise to confirm that it is of an acceptable scientific standard.

## References

[1] : Standards and guidelines for the interpretation of sequence variants: a joint consensus recommendation of the American College of Medical Genetics and Genomics and the Association for Molecular Pathology. *Genet Med* .2015;17(5) : 10.1038/gim.2015.30 405-24 10.1038/gim.2015.30 25741868 PMC4544753

# Reviewer report: Reviewer response for version 2

**DOI:** 10.5256/f1000research.162702.r263441

**Published:** 2024-04-25

**Authors:** James Reecy

**Affiliations:** 1 Department of Animal Science, Iowa State University, Ames, IA, USA

This manuscript reports the search for potential alleles associated with bovine congestive heart failure (BCHF). Using a case-control approach utilizing cattle from the upper plains of western Nebraska, two regions in the genome were identified as potentially containing associated alleles. These regions are centered around ARRDC3 and NFIA. While these alleles can predict risk of disease, they do not fully explain the risk of the disease.

 An interesting issue with this disease is the prevalence of exhibiting the disease with associated with the environment, i.e., elevation. This would indicate a genetic by environmental interaction. Furthermore, there is also a dietary component, i.e., energy density of the diet, of the disease, which was not evaluated in this study.

 While a genetic variant in each of these genes is presented as being associated with risk of the disease, there is no evidence provided that these are causal mutations. Whole genome sequencing data is not presented, thus it is very possible that the causal mutations have yet to be identified. This is a strong possibility given that other cattle populations have a relatively high frequency of these alleles, but do not exhibit the disease incidence to the extent that the studies populations do. There is variant data that is available in freely available databases, e.g., Ensembl. None of these alleles appear to have been tested.

 The authors talk about a validation population of 170 BCHF cases, but nowhere is this population presented in the materials and methods section. Thus, it is unknown if they came from related animals or the same feedlots.

 In Figure 2B, the authors claim that the genetic distances between most of the tested population is very small, except for a few animals, which are clearly separated from the other animals. However, how can the reader know that the observed distances are small compared to other populations when no reference information is provided? A pairwise comparison of the 96 breed samples would have this type of reference.

 The protein data analysis is not presented in the results section of the manuscript.

Is the work clearly and accurately presented and does it cite the current literature?

Yes

If applicable, is the statistical analysis and its interpretation appropriate?

Yes

Are all the source data underlying the results available to ensure full reproducibility?

Yes

Is the study design appropriate and is the work technically sound?

Yes

Are the conclusions drawn adequately supported by the results?

Yes

Are sufficient details of methods and analysis provided to allow replication by others?

Partly

Reviewer Expertise:

I have over 25 years of beef cattle molecular genetics/genomics research experience.

I confirm that I have read this submission and believe that I have an appropriate level of expertise to confirm that it is of an acceptable scientific standard, however I have significant reservations, as outlined above.

# Reviewer report: Reviewer response for version 1

**DOI:** 10.5256/f1000research.120994.r129617

**Published:** 2022-04-26

**Authors:** Cord Drögemüller

**Affiliations:** 1 Institute of Genetics, Vetsuisse Faculty, University of Bern, Bern, Switzerland

The study aims to identify a possible genetic cause for the occurrence of BCHF in North American feedlot cattle. The case control study using high density SNP genotypes revealed significant associations to two different genomic regions. This indicates a possible genetic predisposition, at the same time the presented genotype distributions indicate that it is highly likely a complex inheritance, since the genotypes of the markers in these two genomic regions do not alone explain the emergence of the clinical symptoms.

 The two protein-coding genes annotated in the two mapped chromosomal regions have not been associated with heart disease in humans or animals. In principle, this could be of great biomedical interest, as it is well known that in animal phenotypes we often have the possibility to assign a (new or further) previously unknown function to more or less well characterized genes. Especially the missense variant in the ARRDC3 gene seems to be possibly of pathogenic nature due to the presented conservation of the affected amino acid. What speaks against this is the fact that this variant is obviously not rare in cattle in general, otherwise the SNP would not be present on the commercial 777k array. Table 4 shows impressively that the variant occurs similarly frequently in many other breeds or populations, in all of which there has been no mention of a risk for BCHF. Therefore, because no genome sequence data were available, it is rather to assume that in both cases the identified SNPs are only coupled markers and hardly the molecular cause. This should be clearly differentiated in the paper. It remains unclear whether potentially interesting candidate genes for cardiac dysfunction are located in the vicinity of the two association signals and, given the complex development of the disorder, regulatory variants can be assumed as the cause rather than protein-coding variants, which sometimes lead to phenotypes that manifest themselves at birth or at the beginning of life.

 Overall, the work is partly difficult to read because too many methodological details are listed and, on the other hand, the text often remains vague in the biological interpretation. Therefore, the broadly presented possibility of implementing these first results in practical breeding work seems too offensive to me. In my opinion, an even broader validation study with many more affected animals is needed before these markers are directly considered in selection. The study represents a very interesting first step, but the results should be considered with caution, especially since there is always a residual risk of coupled unknown pleiotropic variants on traits desired for breeding.

 Further comments:

- 'Fedlot cattle' is not a clear term outside of North America, so it would be better to speak of the breed involved (Angus?).
- Were there any other pathological (histological) data on the disease?
- What is known about the possible inheritance of BCHF? Have segregation analyses been performed? What is the heritability?
- If about 5% of feedlot cattle are at risk for BCHF, why have only about 100 affected animals been identified when over 1400000 cattle have been considered? Is the disease rarer than assumed?
- The nomenclature of variants, especially the representation of ARRDC3 alleles, should necessarily follow the current recommendations, see Richards *et al.* 2015 ^1^.
- The intronic variant in the NFIA gene remains functionally absolutely unclear, therefore talk about a linked marker rather than a possible causal variant.
- In the methods section, the races in the panel of 96 unrelated controls should be mentioned.
- The actual linkage disequilibrium between markers in the two associated genomic regions remains unclear in places. In particular, the question arises whether the SNPs mentioned at the end of the methods section are really located with a LD of 1 to the NFIA variant in intron 4 of the gene.
- The wording "homozygous major alleles" on page 10 is unfortunate. Would it not be better to say homozygous for the major allele? An allele itself cannot be homozygous, but the genotype is either hetero- or homozygous for two or one allele.
- In Figure 7 the accession numbers of the listed protein sequences are missing.
- On page 15, a section on the evaluation of the pathogenicity of the missense variant in the ARRDC3 gene is missing next to the presentation of evolutionary conservation. For example, programs such as MutPred2, SIFT or Provean could be used.
- Table 4 would be easier to understand if actual genotype distributions were listed. Also missing is a clear table on the genotype distribution of the two highlighted markers for the animals from the discovery and confirmation cohorts to clearly assess the results. Table 3 is then a further transformation towards possible practical application, but this original publication should include the true genotypes clearly and legibly.
- The second paragraph of the discussion states "an important feature of some BCHF clinical cases..." What exactly does "some" mean here? This should be clarified.

Is the work clearly and accurately presented and does it cite the current literature?

Yes

If applicable, is the statistical analysis and its interpretation appropriate?

I cannot comment. A qualified statistician is required.

Are all the source data underlying the results available to ensure full reproducibility?

Yes

Is the study design appropriate and is the work technically sound?

Yes

Are the conclusions drawn adequately supported by the results?

Partly

Are sufficient details of methods and analysis provided to allow replication by others?

Yes

Reviewer Expertise:

Animal genetics

I confirm that I have read this submission and believe that I have an appropriate level of expertise to confirm that it is of an acceptable scientific standard, however I have significant reservations, as outlined above.

## References

[1] : Standards and guidelines for the interpretation of sequence variants: a joint consensus recommendation of the American College of Medical Genetics and Genomics and the Association for Molecular Pathology. *Genet Med* .2015;17(5) : 10.1038/gim.2015.30 405-24 10.1038/gim.2015.30 25741868 PMC4544753

# Response

**Published:** 2024-02-13

**Authors:** Michael Heaton

**Affiliations:** US Meat Animal Research Center, USA

AUTHORS' RESPONSE TO REVIEWER 2

 The authors appreciate and are grateful for both reviewers’ thoughtful reviews of our article.  Below we have provided point-by-point responses to their suggestions and comments:

**Reviewer 2, point 1:**  “The study aims to identify a possible genetic cause for the occurrence of BCHF in North American feedlot cattle.”

**Authors response 1:**   Our aim has been subtly misstated above, but in an important way.  Our stated aim in the Abstract was “to search for genomic regions associated with this disease”.  Our search was designed to test the null hypothesis that no significant association exists between HD770 SNPs tagging genomic regions and BCHF.  We have indicated throughout the article that we are reporting the association of SNP alleles and not the pathogenic roles for *ARRDC3* and *NFIA* variants associated with BCHF.  Our focus has been on the strength and reproducibility of the detected associations, not their likelihood of being causal.  To underscore our stated aim, we have modified the Introduction section final paragraph to include this text:  “… *to test the null hypothesis that significant association does not exist between haplotype tagging SNPs and BCHF.  Our results rejected the null hypothesis and identified two distinct genomic regions harboring major genetic risk factors associated with BCHF.*”

**Reviewer 2, point 2: ** “Therefore, because no genome sequence data were available, it is rather to assume that in both cases the identified SNPs are only coupled markers and hardly the molecular cause. This should be clearly differentiated in the paper.”

**Authors response 2:**   We agree and have modified the 4 ^th^ and 5 ^th^ sentences of the discussion to state: *“The most significant genome-wide associations were limited to two regions containing ARRDC3 and NFIA genes, respectively.  In each region, multiple linked SNPs were associated with BCHF in feedlot cattle.  It is unknown whether the associated SNPs are merely linked to variants responsible for the elevated BCHF risk, or whether they are involved directly in pathogenesis.“  *As described in our response to Reviewer 1, we underscore this point and make our intentions unambiguous in the last paragraph:   “ *Thus, WGS analysis of the 102 case-control pairs would refine the bounds of the genomic region most strongly associated with BCHF and identify the complete set of tagging SNPs appropriate for use in genotyping by sequencing.  Any novel variants with increased association would potentially represent candidates for functional variants conferring BCHF risk.  A WGS analysis may also identify any newly associated genomic regions previously missed due to ascertainment bias in the SNP selection for the BovineHD BeadChip array.  For these reasons, we are endeavoring to produce and analyze 15-fold WGS coverage from these 204 animals.*” 

**Reviewer 2, point 3:**  “'Fedlot cattle' is not a clear term outside of North America, so it would be better to speak of the breed involved (Angus?).”

**Authors response 3: **  We recognize that the details of North American beef production may not be well known around the world.  Moreover, it varies greatly within the US.   Feedlots may receive all types of beef, dairy, and composite cattle.  Overall, Angus-influenced black cattle represent about 70% of the fattened cattle in the study region.  In the method section we describe a group of 204 calves as 93% solid black, 70% castrated males, and none had visible horns.  Although the cases were defined by clinical presentation, they seemed roughly representative of the feedlots we sampled.  We have inserted the following sentence in the third line of the Introduction section: “ *Feedlot cattle in this region are typically a mix of germplasm derived from popular British and Continental European breeds with more than 60% of the animals having a black hide from Angus contributions.*“

**Reviewer 2, point 4:**   “Were there any other pathological (histological) data on the disease?”

**Authors response 4: **  Systematic histological data was not collected on the 102 pairs of cases and controls.  Tissues were collected from the clinical cases at necropsy, but the tissues of healthy controls were processed into beef and unavailable.

**Reviewer 2, point 5:**  “What is known about the possible inheritance of BCHF? Have segregation analyses been performed? What is the heritability?”

**Authors response 5:**  To date, there have been no reports of inheritance, segregation analysis, or heritability of clinical BCHF.  Also, no other studies have used a BCHF phenotype to directly obtain an estimate that could correspond to our study.  We used a clinically-defined binary phenotype to represent BCHF, specifically, end-stage congestive heart failure mortality in Western Great Plains feedlot cattle.  By comparison all matched pen mate control animals were normal and passed their antemortem inspection at beef processing facilities.  Thus, with only two phenotypic states, the use of variance as a convenient mathematical quantity was not appropriate for estimating heritability.  Moreover, our BCHF study used animals raised in multiple environments and populations, while heritability is a statistical measure defined in the context of a specific environment and a specific population.  For these and other reasons, heritability has not been measured for BCHF.  Since our publication, a report used cardiomegaly observed at slaughter as a proxy for “BCHF” (Buchanan, JW et al., Front. Genet. 2023; 14:1148301).  However, healthy animals with enlarged hearts cattle that have passed antemortem inspection do not meet our criteria for BCHF and would be considered unaffected controls in our study.

**Reviewer 2, point 6:**  “If about 5% of feedlot cattle are at risk for BCHF, why have only about 100 affected animals been identified when over 1400000 cattle have been considered? Is the disease rarer than assumed?”

**Authors response 6: ** The 5% number is the mortality in severely affected pens of cattle.  It was not the percentage of cattle at risk.   To clarify reasons for the case enrollment rate, we have added this text to the Methods section, “ *The case* *enrollment rate of 102 cases per 140,000 feedlot cattle monitored (0.073%) was negatively affected by:  1) removal of sick animals for emergency salvage slaughter, 2) removal of matched unaffected pen mates for beef processing, 3) the rapid onset of congestive heart failure and death, 4) reluctance of overburdened personnel to commit valuable time despite the $500 indemnity offer for each matched pair identified early, and 5) exclusion of necropsied animals meeting some, but not all, of the case definition criteria.* *”*  We also suspect that the disease is underreported due to worker misclassification at necropsy.

**Reviewer 2, point 7:** The nomenclature of variants, especially the representation of ARRDC3 alleles, should necessarily follow the current recommendations, see Richards et al. 20151.

**Authors response 7: ** The Richard et al. 2015  article (“Standards and guidelines for the interpretation of sequence variants”) provides detailed criteria for classifying pathogenic variants in genes known to cause human diseases.  As noted in Authors response 1 above, our stated aim was “to search for genomic regions associated with this disease”.  We are not reporting the pathogenicity of variants in this report.  We have added the rs numbers for the variants highlighted in Table 2 and added context sequence information in a new Table 5.

**Reviewer 2 point 8:** The intronic variant in the NFIA gene remains functionally absolutely unclear, therefore talk about a linked marker rather than a possible causal variant.

**Authors response 8: ** Although it is not clear which sentence in the manuscript that Reviewer 2 is referring, we agree.  Other than the possibility of the *ARRDC3* C182Y missense SNP affecting protein function, all other SNPs associated with BCHF are presumed to be linked variants.  Likewise, it is also possible that the *ARRDC3* C182Y missense SNP is merely linked to a functional variant.  To emphasize this point we have modified this sentence towards the end of the first paragraph of the Discussion section: *Thus, specific DNA sequence variants within ARRDC3 and NFIA are markers for BCHF risk in feedlot cattle in the Western Great Plains, either through linkage to causal variants or direct effects.*

**Reviewer 2, point 9:**  In the methods section, the races in the panel of 96 unrelated controls should be mentioned

**Authors response 9: **  We agree.  This information was in our original submission to F1000Research.  We have restored this content in the Methods section of the revised article.

**Reviewer 2, point 10:** The actual linkage disequilibrium between markers in the two associated genomic regions remains unclear in places.  In particular, the question arises whether the SNPs mentioned at the end of the methods section are really located with a LD of 1 to the NFIA variant in intron 4 of the gene.

**Authors response 10: **  We agree and have rewritten the penultimate paragraph of the article to clarify and discuss the strength of LD between the BCHF-associated SNPs in the two separate genomic regions.  With regards to the two best associated *NFIA* variants spanning 2.32 kb in intron 4, their LD was high (r ^2^ = 0.94).  These two SNPs were only moderately linked to a SNP 127 kb upstream in *NFIA AS2* (r ^2^ = 0.49 and 0.45, Figure S2).

**Reviewer 2, point 11:**  The wording "homozygous major alleles" on page 10 is unfortunate. Would it not be better to say homozygous for the major allele? An allele itself cannot be homozygous, but the genotype is either hetero- or homozygous for two or one allele.

**Authors response 11: **  We agree this was terribly worded.  The sentence on page 10 line 6 now states, “ *Homozygosity for the major allele of either of these two SNPs was associated with increased BCHF risk (OR 7.4, CI _95_, 2.9 to 19,* *Table 2* ).”

**Reviewer 2, point 12:**  In Figure 7 the accession numbers of the listed protein sequences are missing.

**Authors response 12: **  Figure 7 summarizes the results of a much larger ARRDC3 peptide alignment with 80 accession numbers.  We have added this larger alignment with its accession numbers to the extended and underlying data section (Table S2. Extended comparison of ARRDC3 residues near the C182Y position in the Gnathostomes. https://doi.org/10.6084/m9.figshare.22213495

**Reviewer 2, point 13:**  On page 15, a section on the evaluation of the pathogenicity of the missense variant in the ARRDC3 gene is missing next to the presentation of evolutionary conservation. For example, programs such as MutPred2, SIFT or Provean could be used.

**Authors response 13: **  We respectfully disagree.  As emphasized above in Author response 1, our aim here was not to evaluate the pathogenicity of associated SNPs.  In our opinion, such analyses are premature for bead array SNPs, which have a strong selection biased for high minor allele frequencies and represent a only small fraction of the total genetic variation in the region.  In silico predictions of  pathogenicity of missense variants would be more appropriate once WGS for these regions is available, and the full scope of associated variants is known.  However, to increase our understanding of the Y182 variant on the predicted ARRDC3 tertiary structure, we have added alignments from *de novo* AlphaFold2 predicted structure in Figure 9 and introduced a hypothesis for testing (Discussion section).

**Reviewer 2 point 14:**  Table 4 would be easier to understand if actual genotype distributions were listed. Also missing is a clear table on the genotype distribution of the two highlighted markers for the animals from the discovery and confirmation cohorts to clearly assess the results. Table 3 is then a further transformation towards possible practical application, but this original publication should include the true genotypes clearly and legibly.

**Authors response 14: **  We are happy to provide additional lists of genotypes for figures.  We recognize that extracting these genotypes from the provided 770k SNP bead array PLINK file (Supplementary Files S1 through S9) is not trivial.  In addition, the revised article now contains genotypes corresponding to  the 12 SNPs represented Table 2 for the 204 animals (Table S3), the two SNPs from 566 animals represented in Figure 8 (Table S4), and two SNPs for the 1203 animals listed in Table 4 (Table S5). 

 We also note that the homozygous *NFIA* bar chart data in Figure 8 have changed slightly for the 96 Diversity beef bulls and the 96 persistently infected (BVD) feedlot cattle panels.  Their bars increased  due to inadvertent initial use of a 3-SNP *NFIA* haplotypes instead of just the single *NFIA* SNP rs133192205 (ie., BovineHD0300024308 or BCHF32).  Since the 3-SNP *NFIA* haplotypes were not available for the 171 validation cases, we reverted to using only the single NFIA SNP for all cohorts.

**Reviewer 2, point 15:**  The second paragraph of the discussion states "an important feature of some BCHF clinical cases..." What exactly does "some" mean here? This should be clarified.

**Authors response 15: **  We agree.  The sentence has been rephased to read: “More than 50% of the BCHF cases in our study died in the first half of the fattening period.”

# Reviewer report: Reviewer response for version 1

**DOI:** 10.5256/f1000research.120994.r129614

**Published:** 2022-04-20

**Authors:** Alan L. Archibald

**Affiliations:** 1 The Roslin Institute, Royal (Dick) School of Veterinary Studies (R(D)SVS), University of Edinburgh, Midlothian, UK

A thorough analysis of potential associations between genetic factors and susceptibility to bovine congestive heart failure (BCHF) in feedlot cattle is presented. Genetic variants at the ARRDC3 and NFIA loci are shown to be associated with increased risk of BCHF. The SNP variants in these loci do not fully explain the risk of BCHF, nor have causal genetic variants been identified. Genetic tests to identify animals at risk of BCHF and/or at risk of transmitting increased risk to their offspring are proposed.

 The analysis of genetic variation associated with risk of BCHF is limited to SNP variants present on the Illumina HD SNP chip. Given the strong signal associated with the ARRDC3 locus in particular it would have been useful to sequence this locus in multiple individuals of known BCHF phenotype in order to identify candidate causal or risk variants. The authors highlight a missense variant ARRDC3 C182Y for which there is an assay as BovineHD0700027239 on the Illumina HD SNP chip. They note that a cysteine residue is found at this position in  the ARRDC3 protein in a wide range of animals separated by millions of years of evolutionary time.

 Multiple sequence variants have been reported in the bovine ARRDC3 locus including several other missense mutations and premature stop codons as summarised here:

Link

 Several of these missense and premature stop variants are predicted by SIFT analysis to be deleterious whereas the ARRDC3 C182Y variant is not predicted by SIFT analysis to be deleterious.

 Sequence analysis of the ARRDC3 locus in individuals of known BCHF phenotypes might reveal candidate causal or risk variants.

 In order to link the SNP information reported in the manuscript more readily to other data sources it would be useful to add rs numbers. For example, the rs number for the ARRDC3 C182Y variant for which there is an assay as BovineHD0700027239 on the Illumina HD SNP chip is rs109901274.

 The frequencies of the ARRDC3 risk alleles as summarised in Table 4 are very high in some breeds, including Angus, Red Angus, Murray Grey and i) pose a challenge to selective breeding in these breeds to reduce the risk of BCHF and ii) raises questions about why the incidence of BCHF is not higher.

Is the work clearly and accurately presented and does it cite the current literature?

Yes

If applicable, is the statistical analysis and its interpretation appropriate?

I cannot comment. A qualified statistician is required.

Are all the source data underlying the results available to ensure full reproducibility?

Yes

Is the study design appropriate and is the work technically sound?

Yes

Are the conclusions drawn adequately supported by the results?

Yes

Are sufficient details of methods and analysis provided to allow replication by others?

Yes

Reviewer Expertise:

Farmed animal genetics and genomics

I confirm that I have read this submission and believe that I have an appropriate level of expertise to confirm that it is of an acceptable scientific standard.

# Response

**Published:** 2024-02-13

**Authors:** Michael Heaton

**Affiliations:** US Meat Animal Research Center, USA

AUTHORS' RESPONSE TO REVIEWER 1

 The authors appreciate and are grateful for both reviewers’ thoughtful reviews of our article.  Below we have provided point-by-point responses to their suggestions and comments:

**Reviewer 1, point 1:**  “Sequence analysis of the *ARRDC3* locus in individuals of known BCHF phenotypes might reveal candidate causal or risk variants.”

**Authors response 1:**   We agree. We attempted to indicate this point in the last paragraph of the discussion.  However, to further underscore this point and make our intentions unambiguous, we have revised the first paragraph of the discussion: “ *It is unknown whether the associated SNPs are merely linked to variants responsible for the elevated BCHF risk, or whether they are involved directly in pathogenesis.  Whole genomic sequence (WGS) analyses are underway for the 102 case-control pairs of animals and may unlock further insights in the future.*”   The last paragraph of the discussion was also revised to emphasize this point:  “ *Any novel variants with increased association would potentially represent candidates for functional variants conferring BCHF risk.  A WGS analysis may also identify any newly associated genomic regions previously missed due to ascertainment bias in the SNP selection for the BovineHD BeadChip array.  For these reasons, we are endeavoring to produce and analyze 15-fold WGS coverage from these 204 animals.*” 

**Reviewer 1, point 2**: “In order to link the SNP information reported in the manuscript more readily to other data sources it would be useful to add rs numbers.”

**Authors response 2:**   We agree and have added rs numbers to Table 2 for the 12 SNPs most associated with BCHF.  To provide additional unique molecular information defining these 12 SNP, we also added Table 5 which includes 200 bp of adjacent context nucleotide sequence from the ARS-UCD2.1 bovine reference assembly, together with flanking SNP variants greater than 0.015 frequency as identified in a 96-member beef cattle diversity panel.  Together, these details are important for designing accurate assays to score genotypes at SNP sites associated with BCHF in feedlots of the Western Great Plains.

**Reviewer 1, points 3 and 4: ** “The frequencies of the ARRDC3 risk alleles as summarised in Table 4 are very high in some breeds, including Angus, Red Angus, Murray Grey and i) pose a challenge to selective breeding in these breeds to reduce the risk of BCHF and ii) raises questions about why the incidence of BCHF is not higher.”

**Authors response 3 and 4: ** We agree and have added two sequential paragraphs to the Discussion section:  “ *The high frequencies of ARRDC3 and NFIA risk alleles in some affected breeds (> 0.65) raise the question of why the disease prevalence is relatively low even in outbreaks (> 0.07)?  This phenomenon may be due to the multifactorial nature of complex disease.  The development of BCHF in cattle is presumed to be influenced by exposure to non-genetic factors, such as elevation (hypoxia), extreme weather conditions, feeding schedules, ionophores, feed grade antibiotics, steroid hormone implants, and beta-adrenergic agonist feed additives (Krafsur et al, 2019, Neary et al. 2018).  These environmental and management factors may play significant but unknown roles in disease development.  In addition, most complex diseases are considered polygenic with variation at many genes having small effects (Khera et al. 2018).  DNA sequence variation identified here in ARRDC3 and NFIA genomic regions may predispose cattle to BCHF under certain conditions, despite the absence of any known causal pathogenic mutations.*

*The high frequencies of risk alleles observed in some breeds also poses a challenge for selective breeding.  Although estimates for the positive predictive value of the ARRDC3 and NFIA markers were low (0.10), the negative predictive value was quite high (0.99). Consequently, identifying breeding stock lacking these risk factors could prove useful for reducing a herd's overall risk.  The challenge is finding sires without the ARRDC3 and NFIA risk alleles in breeds with a high prevalence of high-risk alleles.  For instance, the largest affected breed in the United States, American Angus, has more than 300,000 bulls registered annually in its association.  However, less than 5% (15,000) are expected to possess the optimal low-risk genotypes at both the ARRDC3 and NFIA gene regions (M. Heaton, L. Kuehn, and B. Vander Ley 2022, unpublished). To identify the best bulls for decreasing BCHF risk alleles in their herds, cattle producers may need to obtain genotypes from hundreds of candidate sires. Unfortunately, this information is not widely available for most bulls at present.  Despite these challenges, there are a number of reasonable selection strategies that could help reduce the risk of BCHF in the long term, depending on the specific goals and circumstances of the cattle producer.* “
